# Recombinase-Controlled Multiphase Condensates Accelerate Nucleic Acid Amplification and CRISPR-Based Diagnostics

**DOI:** 10.1021/jacs.4c11893

**Published:** 2025-02-13

**Authors:** Aimorn Homchan, Maturada Patchsung, Pheerawat Chantanakool, Thanakrit Wongsatit, Warunya Onchan, Duangkamon Muengsaen, Thana Thaweeskulchai, Martin Tandean, Theeradon Sakpetch, Surased Suraritdechachai, Kanokpol Aphicho, Chuthamat Panchai, Siraphob Taiwan, Navin Horthongkham, Taweesak Sudyoadsuk, Aleks Reinhardt, Chayasith Uttamapinant

**Affiliations:** † School of Biomolecular Science and Engineering, Vidyasirimedhi Institute of Science and Technology (VISTEC), Rayong 21210, Thailand; ‡ Department of Microbiology, Faculty of Medicine Siriraj Hospital, Mahidol University, Bangkok 10700, Thailand; § Frontier Research Center, Vidyasirimedhi Institute of Science and Technology (VISTEC), Rayong 21210, Thailand; ∥ Yusuf Hamied Department of Chemistry, University of Cambridge, Cambridge CB2 1EW, United Kingdom

## Abstract

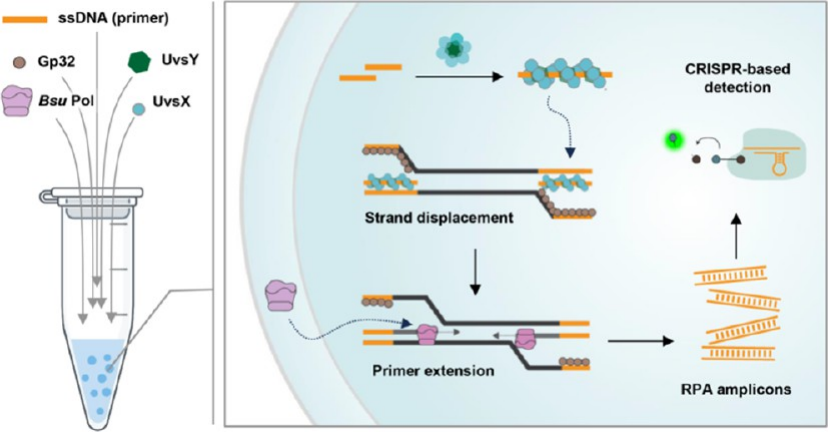

Isothermal techniques for amplifying nucleic acids have found extensive applications in genotyping and diagnostic tests. These methods can be integrated with sequence-specific detection strategies, such as CRISPR-based detection, for optimal diagnostic accuracy. In particular, recombinase-based amplification uses proteins from the Escherichia virus T4 recombination system and operates effectively at moderate temperatures in field and point-of-care settings. Here, we discover that recombinase polymerase amplification (RPA) is controlled by liquid–liquid phase separation, where the condensate formation enhances the nucleic acid amplification process. While two protein components of RPA could act as scaffold proteins for condensate formation, we identify T4 UvsX recombinase as the key regulator orchestrating distinct core–shell arrangements of proteins within multiphase condensates, with the intrinsically disordered C-terminus of UvsX being crucial for phase separation. We develop volumetric imaging assays to visualize RPA condensates and the reaction progression in whole volumes, and begin to dissect how macroscopic properties such as size distribution and droplet count could contribute to the overall reaction efficiency. Spatial organization of proteins in condensates may create optimal conditions for amplification, and disruption of such structures may diminish the amplification efficiency, as we demonstrate for the case of reverse transcription-RPA. The insight that RPA functions as a multiphase condensate leads us to identify the UvsX^D274A^ mutant, which has a distinct phase-separation propensity compared to the wild-type enzyme and can enhance RNA detection via RPA-coupled CRISPR-based diagnostics.

## Introduction

Cellular DNA repair and replication require strategies to temporarily unwind the DNA duplex to allow the excision of lesions or sequence-specific synthesis. Such strand exchange can be catalyzed by helicases and recombinases at physiological temperatures. In biotechnology, these enzymes can replace cycles of thermal denaturation and annealing achieved with thermal cyclers in a polymerase chain reaction (PCR) and help amplify nucleic acids at single near-physiological temperatures. Such isothermal amplification reactions—which include helicase-dependent amplification (HDA),^1^ SSB-Helicase Assisted Rapid PCR (SHARP),^2^ Recombinase Polymerase Amplification (RPA/RAA),^3^ and Strand Invasion Based Amplification (SIBA)^4^—have found wide uses in genotyping and diagnostic assays, particularly for point-of-care and field settings where energy sources and equipment are limited. Isothermal amplification technologies can be coupled to sequence-specific detection approaches, particularly clustered regularly interspaced short palindromic repeats (CRISPR)-based detection,^5,6^ to further increase detection sensitivity and specificity of genetic targets to the point of clinical utility, as demonstrated in recent years.^7−12^

**Figure 1:**
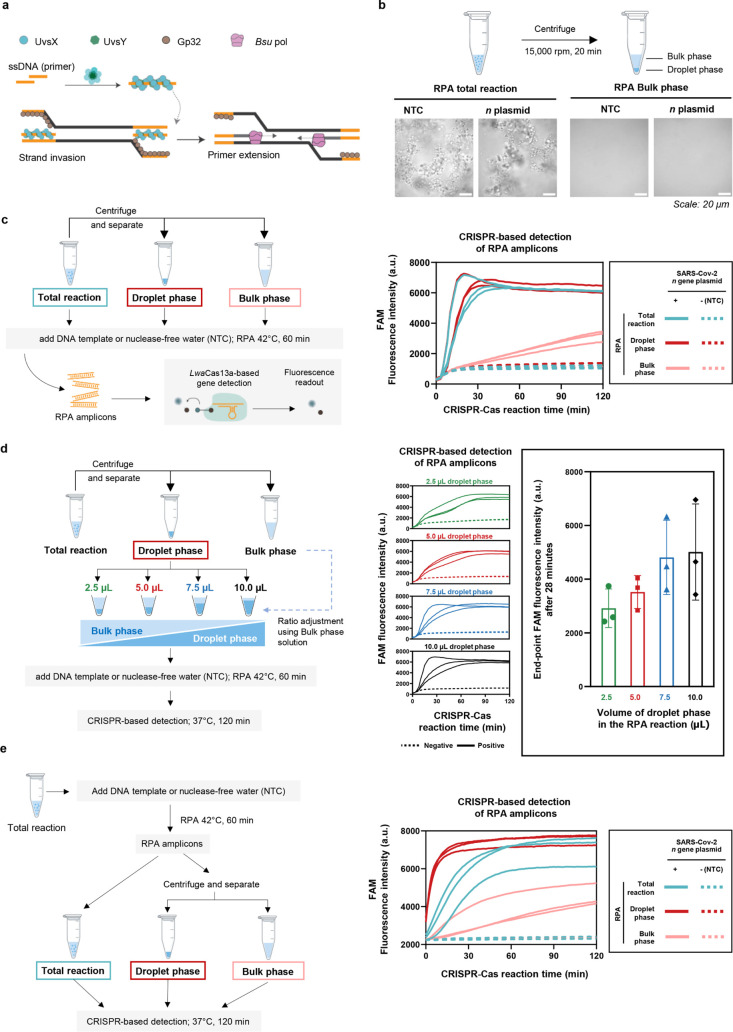
Recombinase-mediated DNA amplification undergoes liquid–liquid phase separation and is accelerated in the droplet phase. (a) The Recombinase Polymerase Amplification (RPA) process. UvsX recombinase, assisted by UvsY, binds to primers and catalyzes strand invasion at complementary sequences. The displaced DNA strand is bound and stabilized by the single-stranded DNA-binding protein Gp32. Subsequently, the UvsX–UvsY recombinase complex disassembles, leaving the primers accessible to chain extension by the strand-displacing *Bsu* DNA polymerase (*Bsu* Pol). (b) Representative brightfield images of the total RPA reaction and bulk solution after centrifugation. The RPA reaction was centrifuged to separate bulk and droplet phases. The resulting solution underwent RPA amplification before mounting on a glass dish for visualization under a confocal microscope. Scale bar, 20 μm. (c) LwaCas13a-based *n* gene of SAR-CoV-2 detection of amplified RPA products produced by the separated droplet and bulk phase, compared to the total RPA reaction (left). Kinetic traces of FAM fluorescence generation from three replicates of each condition are shown (right). (d) Titration of increasing amount of the isolated droplet phase to the RPA reaction. Following the preparation of the RPA reaction, aliquots were taken to represent the total reaction. The remaining RPA reaction mixture was centrifuged to separate the bulk and droplet phases. The droplet phase solution was then added to the bulk phase according to the volumes specified (left). Each condition then underwent RPA amplification. Kinetic traces of FAM fluorescence generation (middle) and end-point fluorescence intensities at the 28-min CRISPR-Cas reaction time (right) from three replicates of each condition are shown. Error bars, ± s.d. (e) DNA amplicons of RPA remain associated with the droplet phase. RPA was performed before centrifugal separation of the droplet and bulk phases (left). RPA amplicons in each phase along with the total reaction were then detected via CRISPR-Cas13a. Kinetic traces of FAM fluorescence generation from three replicates of each condition are shown (right). For (b–e) RPA was initiated by the addition of 10,000 copies of the pUC57–2019-nCoV-N plasmid as DNA template; and negative controls (NTC) used RNase-free water *in lieu* of DNA input.

Among isothermal amplification reactions, recombinase polymerase amplification (RPA) is highly efficient at mesophilic temperatures (37–42 °C) and uses four core protein components from mesophilic organisms: the large fragment of DNA polymerase I from *Bacillus subtilis* (*Bsu* Pol) or *Staphylococcus aureus* Pol I and UvsX, UvsY, and Gp32 from Escherichia virus T4. All four proteins have been extensively biochemically characterized,^13−22^ and the mechanism of RPA is postulated based on the functions of individual proteins, as follows (Figure 1a): (1) UvsX recombinase forms presynaptic filaments on single-stranded DNA (such as primers) with initial nucleation from UvsY recombinase loader; (2) the UvsX-primer complex actively searches for homologous sequences and, once located, performs strand exchange; (3) Gp32 ssDNA-binding protein is proposed to stabilize the unwound DNA strand; and (4) the primer is extended by strand-displacing *Bsu* Pol. Beyond detection and diagnostic applications, since RPA uses three core proteins from the recombination machinery of the T4 phage, it can additionally serve as a biologically relevant *in vitro* model for genetic recombination, particularly for homologous pairing and strand exchange stages.

Despite its wide use and many advantages, RPA is not without limitations. First, RPA primers are longer than PCR primers to provide sites for recombinase binding, but in turn, produce more nonspecific binding, reducing detection sensitivity and specificity, especially for multiplexed amplification. Second, RPA only works well in generating short amplicons (ideally <200 bp), despite the decent processivity of *Bsu* Pol. Engineered DNA polymerases with improved processivity did not improve RPA,^23^ suggesting that other factors beyond Pol processivity govern the reaction. Third, when coupled with reverse transcriptase to amplify RNA targets, reverse-transcription (RT)-RPA has worse efficiency compared to conventional RPA.^24^ Fourth, due to its complex and proprietary formulations, RPA cannot be easily prepared in laboratories and is rarely, if ever, used in biochemical studies of the recombination process, where precise manipulations of the reaction components are required.

We recently reported our own formulations of recombinase-mediated amplification^9^ to enable its easier access in low- and middle-income countries, particularly for low-cost diagnostics. The in-house production further enabled us to probe how RPA functions and potentially improve the reaction. Our formulations of RPA and commercial RPA rely on the same set of core proteins, so their mechanisms are likely similar.

We postulated that condensate formation via liquid–liquid phase separation (LLPS) may govern *in vitro* recombinase-mediated DNA amplification, based on the following prior evidence. First, RPA requires a crowding agent such as Carbowax20 or PEG20000^3^, which is known to promote condensate formation via the macromolecular crowding effect for good amplification efficiencies. We verified that the requirement for a crowding agent also holds for our own-formulated RPA (Figure 1a,b). Second, proteins similar to those used in the RPA reaction—particularly *Escherichia coli* SSB^25^ and Rad52,^26^ the eukaryotic functional equivalent of UvsY—are known to undergo phase separation. Third, many of the most well-characterized phase-separating proteins, including fused-in-sarcoma (Fus),^27,28^ TDP-43,^29,30^ 53BP1,^31^ and Rad52,^26^ are regulators of DNA double-strand break repair and can promote or inhibit homologous recombination, highlighting LLPS as a key mechanism to modulate the genetic recombination machinery. Known biological condensate systems rely on weak multivalent biomolecular interactions and can improve reaction specificity, accelerate reaction kinetics, or suppress reactions and pathways. In the case that RPA can undergo liquid–liquid phase separation, it is unclear how the process affects RPA efficiency. Having this understanding would pave the way for improving RPA, particularly by altering the properties of the condensates.

Herein, we show that recombinase-mediated nucleic acid amplification indeed undergoes phase separation and that the condensate formation accelerates the reaction. While two protein components of RPA can act as scaffold proteins for condensate formation, we identified UvsX as the dynamic master regulator of protein organization within condensates. Protein components within RPA condensates form distinct core–shell multiphase arrangements, and spatial separation between UvsX and *Bsu* Pol seems to optimize their activity within the RPA reaction. Through volumetric imaging, we characterized the total droplet number and size distributions of RPA droplets across conditions and began to establish the relationship between the physical properties of RPA droplets and RPA reaction efficiency. The insight that RPA is accelerated within condensates leads us to further improve the reaction for nucleic acid amplification and CRISPR-based diagnostics through recombinase engineering, which modulates the phase-separation propensity of the protein.

## Results

### Recombinase-Mediated DNA Amplification Undergoes LLPS and is Accelerated in the Droplet Phase

First, we checked whether commercial RPA could undergo phase separation. We reconstituted RPA by adding the rehydration buffer to the lyophilized TwistAmp Basic reagent and mixing well before observing the resulting mixture on a brightfield microscope. We could readily observe droplets of varying sizes (Figure 1b, left). The droplets were formed without the need to add the template plasmid, primers, or magnesium acetate, suggesting that the condensate formation is independent of nucleic acids and the nucleic-acid-dependent activities of RPA protein components. The condensates persisted even with mechanical agitation (Figure S2a) so they are likely to be present in standard RPA setups where agitation is recommended to promote amplicon diffusion, increase interactions of RPA components, and enhance the reaction (Figure S2b).

We next assessed whether DNA amplification by RPA is enhanced in the droplet phase. In bulk activity measurements, we separated the denser, condensate-containing “droplet” phase of the RPA reaction from the remaining condensate-free “bulk” supernatant phase (Figure 1b, right) by centrifugation and compared the RPA activity in both separated phases to the total, condensate-containing reaction. RPA activity was measured via sensitive *Leptotrichia wadei* Cas13a (LwaCas13a)-based RNA detection,^6^ which required conversion of DNA amplicons to RNA via *in vitro* transcription (Figure 1c). RNA target recognition by crRNA-programmed LwaCas13a triggers its collateral activity, resulting in the cleavage of a quenched FAM-RNA reporter in the reaction to elicit FAM fluorescence. Kinetics of FAM fluorescence signal generation of CRISPR-Cas13a reactions were much faster when the droplet phase or the total RPA reaction was used as input compared to when the supernatant was used (Figures 1c and S3), suggesting that the condensates removed from the supernatant majorly contributed to the RPA activity. We titrated the amount of the isolated droplet phase to the isolated bulk phase, initiated RPA by adding in DNA template, and then performed CRISPR-Cas13a reactions to detect the resulting amplicons from RPA. We found that increasing the amount of the isolated droplet phase resulted in faster FAM fluorescence signal generation from CRISPR-Cas13a reactions, suggesting higher RPA efficiency when more of the isolated droplet phase was used (Figure 1d).

We further confirmed that condensate removal did not cause significant changes to protein concentrations in the supernatant, bulk phase of RPA (Figure S4a), suggesting that the loss of protein content is not the reason RPA activity dropped when condensates are removed. Resulting DNA amplicons from RPA seemed to remain associated with the droplet phase, as centrifugation to separate the bulk and droplet phases after RPA reactions had completed showed a higher amplicon presence in the droplet phase, as assessed by DNA concentration measurements (Figure S4b) and CRISPR-Cas13a reactions (Figure 1e).

### Monitoring DNA Amplification in Individual Condensates of RPA

**Figure 2:**
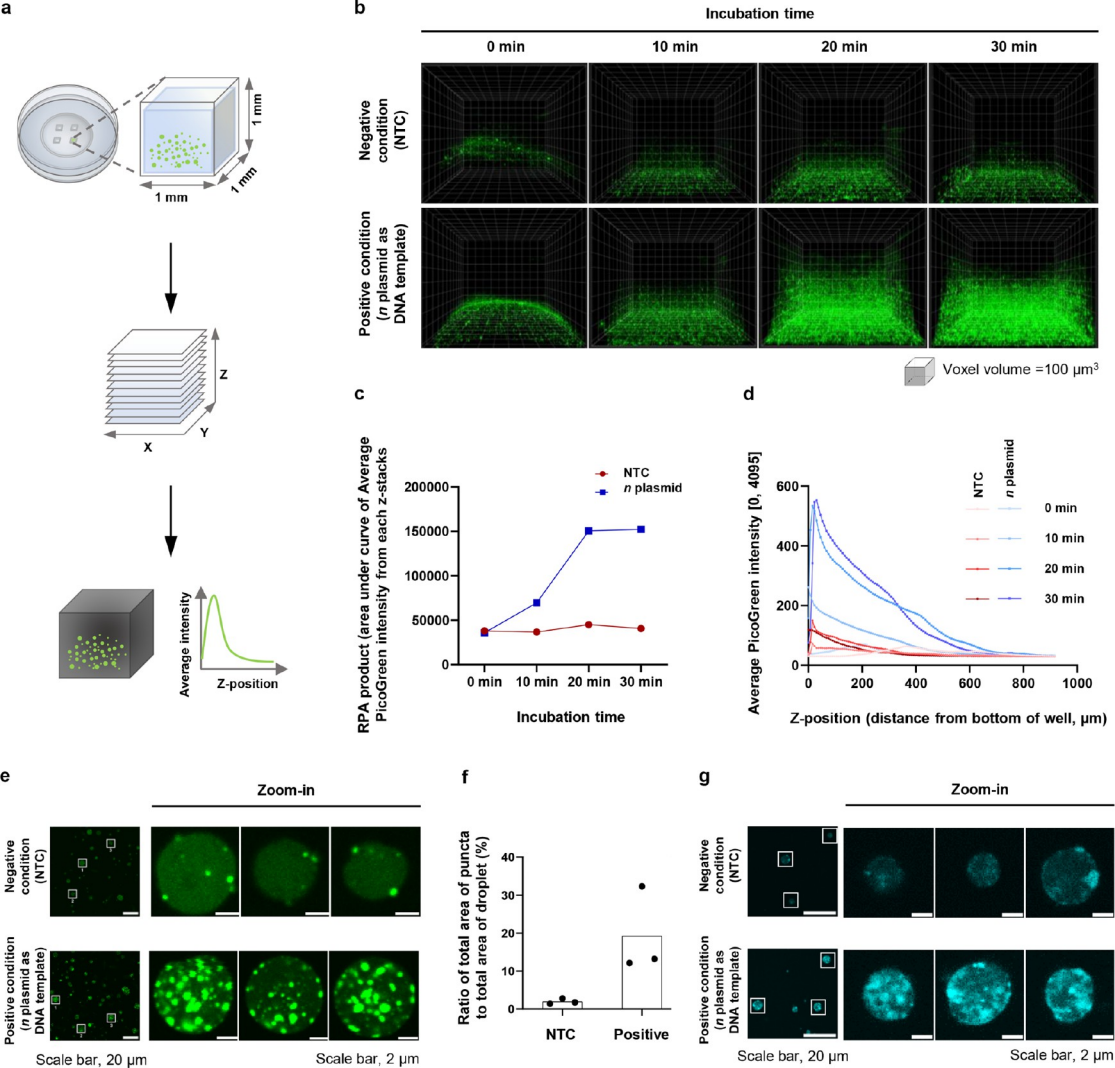
Monitoring DNA amplification in individual RPA condensates DNA synthesis in RPA droplets, using the SARS-CoV-2 *n* plasmid as DNA template for RPA, was monitored by PicoGreen dsDNA-staining dye at different reaction time points (0–30 min). For each time point, 9 μL of RPA samples were combined with 1 μL of PicoGreen staining solution. The resulting mixture was visualized under a fluorescence confocal microscope. (a) Overview of the 3D imaging workflow. To visualize RPA products, 1 μL of the stained mixture was loaded into a custom-fabricated 1 mm^3^ well on an imaging dish and imaged using a confocal microscope equipped with a 10× objective lens and 488 nm laser excitation. *Z*-series images were collected from the bottom to the top of the well to reconstruct a 3D-view of PicoGreen-positive droplets in the RPA reaction. (b) Representative 3D images of PicoGreen-stained RPA droplets. Images from reaction time points of 0, 10, 20, and 30 min are shown for the *n* plasmid condition, and a no-template negative control condition. Voxel volume, 100 μm^3^. (c) Quantification of PicoGreen staining of RPA droplets over time. Combined areas under the curve (AUC) of mean PicoGreen intensities from each *Z*-stack were plotted against RPA reaction times. (d) Mean PicoGreen fluorescence intensity as a function of *Z*-position (distance from the bottom of the well, μm). An additional replicate for (b-–d) is shown in Figure S5. (e) High-magnification imaging with a 100× oil immersion objective of PicoGreen-stained RPA droplets. Images for the template (*n* plasmid)-containing reaction and the no-template negative control are shown. (f,) Quantification of the ratio between the area occupied by PicoGreen puncta and the total droplet area. Data from three puncta from each condition (positive amplification vs negative control) are shown. Details of quantification are shown in Figure S6. (g,) Imaging of DNA synthesis in RPA droplets with Hoechst 33342. An RPA reaction was allowed to proceed for 30 min, then stained with 1.5 μg/mL Hoechst 33342 before confocal imaging. Images for the template (*n* plasmid)-containing reaction and the no-template negative control are shown.

We tested different DNA staining dyes for their abilities to detect RPA amplicons within individual condensates and found PicoGreen to be optimal, likely due to its strong preference for dsDNA over other forms of nucleic acids. We used two magnifications to image the droplets under confocal microscopy: volumetric imaging at lower, 10× magnification to capture PicoGreen signals from whole reaction volumes, and cross-sectional imaging at higher, 100× magnification to assess distributions of amplicons within individual droplets. To accomplish the former, we created customized PDMS imaging chambers in which we added ∼1 μL of RPA reactions combined with PicoGreen before performing 3D confocal imaging (Figure 2a). We observed increased PicoGreen puncta over longer times of RPA reactions, indicative of successful DNA amplification, which increased stainable amplicons over time; negative controls with the DNA template omitted, in contrast, showed relatively constant background through different RPA reaction times (Figures 2b,c and S5). Analyses of the Z-positions of PicoGreen signals indicated the accumulation of the dye at the bottom of the well, consistent with dye staining of DNA in dense condensates sedimented at the bottom of the well (Figure 2d).

At high magnification, we observed numerous PicoGreen puncta within RPA droplets, covering ∼20% of droplet areas, only when DNA synthesis was initiated in the RPA reaction (Figures 2e,f and S6). We speculated that localized PicoGreen puncta could be due to localized DNA amplification because of the limited movement of the DNA template and the resulting amplicons within condensates. We also successfully stained DNA amplicons inside condensates with Hoechst 33342 dye, albeit with a weaker signal-to-noise ratio compared to PicoGreen (Figure 2g). Concentrated DNA puncta, potentially indicative of localized amplification within condensates, were also observed with Hoechst.

Taking together the bulk and the individual droplet measurements of RPA activity, we concluded that DNA amplification by RPA is greatly accelerated in the droplet phase of RPA.

### UvsX and Gp32 as Scaffold-Like Proteins for Condensate Formation

**Figure 3:**
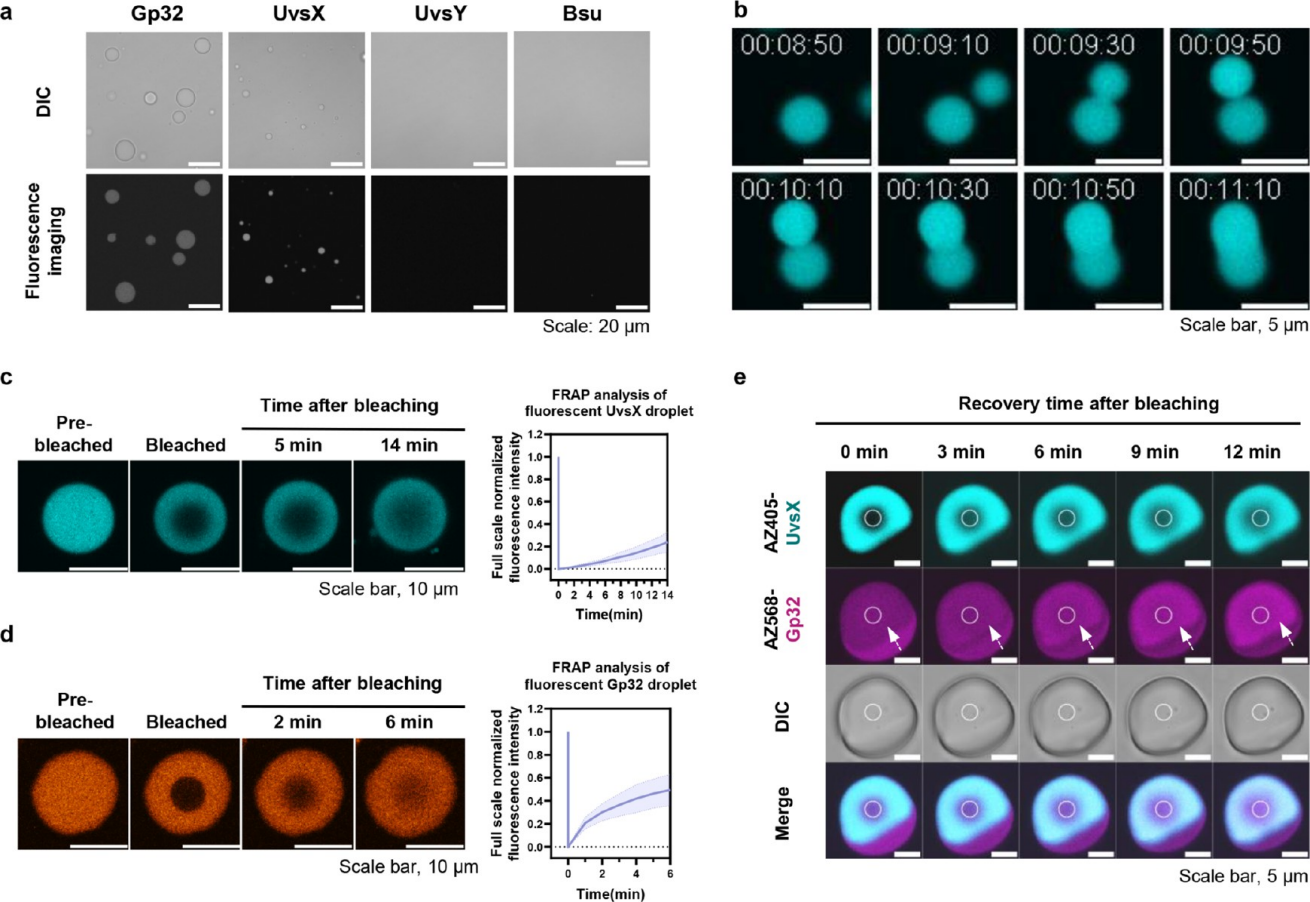
UvsX and Gp32 can individually form condensates **a**, Phase separation of individual proteins of RPA. Confocal fluorescence images of mixtures between unlabeled and fluorescently labeled protein components of RPA are shown. The concentrations of the mixed proteins are as follows: 2.1 μM UvsX, 0.2 μM AZ647-labeled UvsX; 2.2 μM UvsY, 0.2 μM AZ488-labeled UvsY; 26.4 μM Gp32, 0.2 μM AZ568-labeled Gp32; and 2.2 μM *Bsu* Pol, 0.2 μM AZ647-labeled *Bsu* Pol. The mixtures were prepared in RPA buffer (50 mM Tris-HCl pH 7.5, 100 mM KOAc, 14 mM Mg(OAc)_2_, 2 mM DTT, 5% (w/v) PEG20000). Scale bar, 20 μm. **b,** Fusion dynamics of UvsX droplets. Time series of confocal images obtained upon mixing 3.3 μM UvsX with 0.2 μM AZ405-labeled UvsX in RPA buffer showed fusion of droplets. Images were shown at 20-s intervals. Scale bar, 5 μm. **c,** FRAP of UvsX droplets. FRAP was performed using 6 μM UvsX with 0.4 μM AZ405-labeled UvsX (bleached area diameter: 5 μm) (left). After bleaching fluorophores at the center of the droplet, fluorescence intensity recovery was tracked over time. Fluorescence in the bleached region recovered to ∼24% of the initial intensity at the prebleached state by 14 min (right). **d,** FRAP of Gp32 droplets. FRAP was performed using 26 μM Gp32 with 0.4 μM AZ568-labeled Gp32 (bleached area diameter: 5 μm) (left). Fluorescence in the bleached region recovered to ∼49% of the initial intensity at the prebleached state by 5 min (right). **e**, Two-color FRAP of UvsX-Gp32 multiphase droplet. Samples were prepared with 6 μM UvsX and 0.3 μM AZ405-labeled UvsX, along with 26 μM Gp32 and 0.3 μM AZ568-labeled Gp32. Following bleaching of fluorophores at the center of the droplet (bleached area diameter: 5 μm), the recovery of fluorescence intensities was monitored over time. The migration of Gp32 was highlighted by white arrows.

To further investigate the driver of phase separation of RPA, we prepared fluorescently tagged protein components of RPA to high purity via succinimidyl ester-mediated amide coupling (Figure S7). To assess the phase-separation capability of individual RPA proteins, each labeled protein was mixed with its unlabeled counterpart at a labeled-to-unlabeled protein ratio of ∼5:95, and the total concentration of the protein was kept at concentrations relevant to RPA (3.3 μM UvsX; 3.3 μM UvsY; 26 μM Gp32; and 1.8 μM *Bsu* Pol) for confocal imaging. While none of the proteins phase-separated to form condensates under PEG-free buffer conditions (50 mM Tris pH 7.5, 100 mM potassium acetate, 2 mM DTT, and 14 mM magnesium acetate), UvsX and Gp32 readily formed condensates under RPA-like conditions in which the same buffer was supplemented with 5% w/v PEG20000 (Figures 3a and S8). While Gp32 phase-separated in a DNA-independent manner and at critical concentrations similar to its bacterial counterpart *E. coli* SSB,^25^ phase separation of UvsX in the absence of presynaptic filament formation on DNA was surprising and prompted our further investigations. UvsX condensates displayed dynamic behavior and readily fused, contributing to their growth in size at higher protein concentrations (Figure 3b). However, their behavior under fluorescence recovery after photobleaching (FRAP) experiments suggested the droplets were more gel-like, with <10% fluorescence recovery in 6 min (and 24% recovery at 14 min) in comparison to 49% fluorescence recovery observed in liquid-like Gp32 droplets in 6 min (Figure 3c,d). Fluorescence recovery within the bleached regions of the UvsX/Gp32 mixture indicated a faster movement of Gp32 compared with UvsX within the core phase (Figure 3e).

### UvsX is the Master Organizer of liquid–liquid Phase Separation of RPA

**Figure 4:**
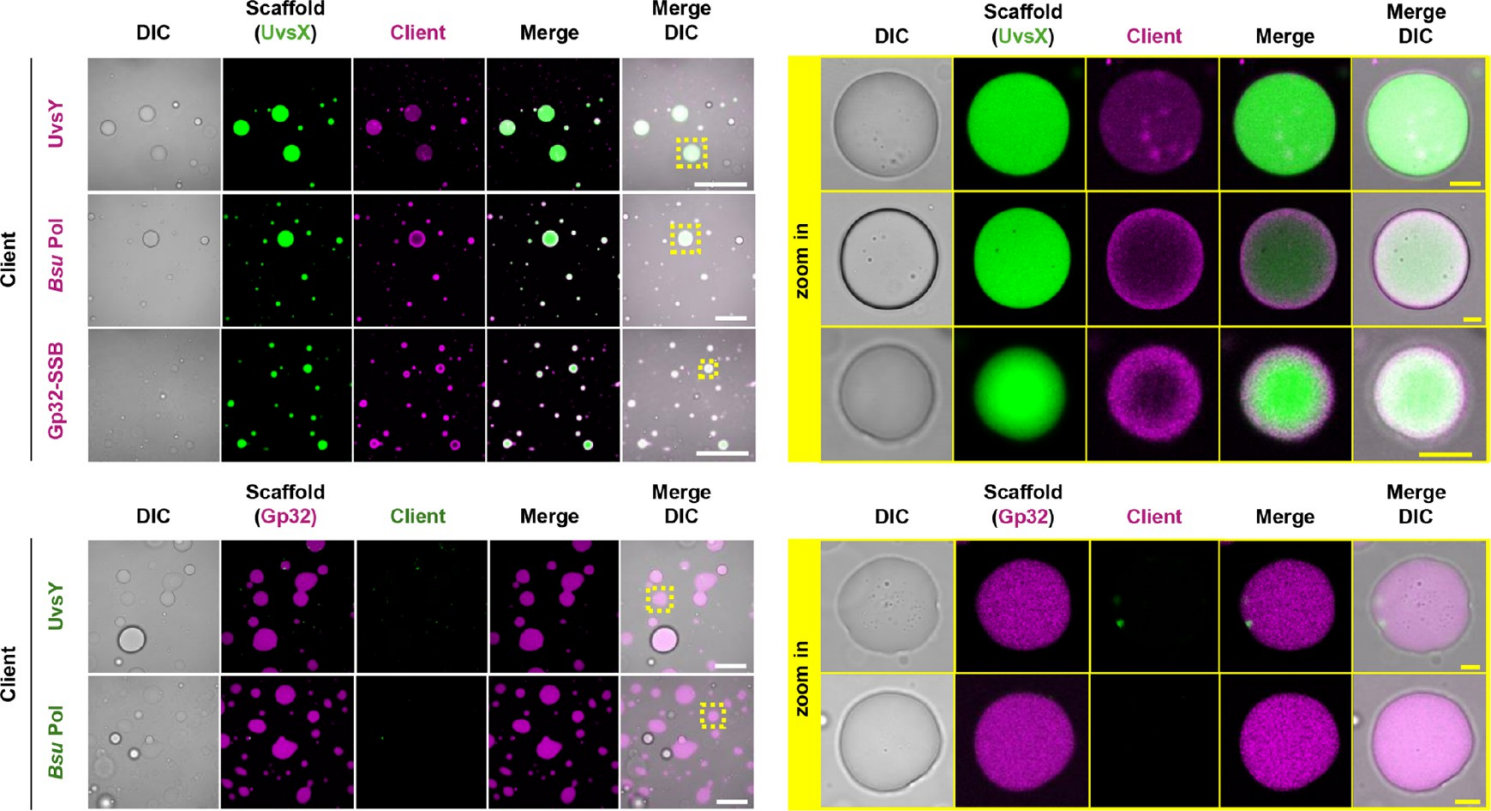
Pairwise imaging of RPA proteins in condensates Scaffold proteins UvsX and Gp32, both capable of forming condensates, were combined with other RPA proteins. The concentrations of the mixed proteins mimic those found in the RPA reaction: 3.3 μM UvsX with 0.2 μM AZ405-labeled UvsX; 3.3 μM UvsY with 0.2 μM AZ488-labeled UvsY; 26 μM Gp32 with 0.2 μM AZ568-labeled Gp32; and 1.8 μM *Bsu* Pol with 0.2 μM AZ647-labeled *Bsu* Pol. This composite mixture is prepared in an RPA buffer containing 50 mM Tris-HCl pH 7.5, 100 mM KOAc, 14 mM Mg(OAc)_2_, 2 mM DTT, and 5% (w/v) PEG20000. For all pairwise combinations, zoom-in images of the boxed droplets are also shown. Scale bars, 50 μm (left panel) and 5 μm (right panel for zoom-ins). Images are representative of three replicates. Additional views are in Figure S9.

UvsX and Gp32 can independently undergo phase separation, indicating their potential roles as scaffold proteins for other “clients” in droplet formation. We thus asked if they could recruit other RPA proteins as clients within droplets. Such recruitment of biomacromolecules eliminates solvents and increases intermolecular contacts and may explain the RPA reaction acceleration we see within the droplet phase. We performed pairwise imaging of one scaffold protein (UvsX or Gp32) and one potential client protein (UvsY, *Bsu* Pol) under RPA-like conditions; we also imaged the two scaffold proteins (UvsX and Gp32) together under similar conditions (Figures 4 and S9).

While both UvsX and Gp32 still phase-separated in the presence of other RPA proteins, we found that only UvsX, not Gp32, can recruit both UvsY and *Bsu* Pol to droplets, suggesting UvsX’s role as the master organizer of LLPS of RPA. UvsX and UvsY, which function cooperatively in homologous recombination within genetic exchange and DNA repair pathways, formed relatively homogeneous droplets upon phase separation. UvsX and Gp32, which can independently form droplets, form biphasic condensates when mixed together, with UvsX consistently forming the core layer and Gp32 the outer shell layer. The precise organization of UvsX and Gp32 in biphasic condensates is likely linked to their innate physicochemical properties, with the core UvsX layer having higher interfacial free-energy densities^32^ resulting from the layer being more hydrophobic^33^ than the Gp32 shell. The enhanced hydrophobicity of the UvsX core layer may be relevant to its DNA strand exchange activity, which relies on electrostatic contacts and therefore would be favored in a more hydrophobic environment.

Although *Bsu* Pol is unable to undergo phase separation on its own, it can be recruited to UvsX droplets. UvsX always forms the core layer and *Bsu* Pol the shell, suggesting the presence of sufficient heterotypic interactions between UvsX and *Bsu* Pol to stabilize the organization, as well as the relatively higher hydrophobicity of the UvsX core layer. Unlike the thick Gp32 shell, the *Bsu* Pol shell layer appears as a thin, almost two-dimensional wetting layer instead of a proper phase, indicating little-to-no homotypic interactions between *Bsu* Pol macromolecules, consistent with the lack of independent phase separation of *Bsu* Pol.

While UvsX appears as the core and Gp32 as the shell in the initial organization of UvsX-Gp32 biphasic condensates, the protein organization over time within condensates is highly dynamic, and the core–shell organization is not strictly maintained. In the UvsX/Gp32 FRAP experiment, Gp32 appeared more in the core layer over time (Figure 3e). These observations suggest that Gp32 exhibits a more dynamic exchange with the surroundings through the phase boundary than UvsX, a property within condensates that may explain a crucial role of Gp32 as a secondary strand exchange mediator in an RPA reaction. Outside of RPA, Gp32 is known to facilitate strand invasion by itself and can work with *Bsu* Pol in an isothermal DNA amplification reaction,^34^ without involvement from liquid–liquid phase separation.

### Phase Separation of UvsX is Mediated by Its C-Terminal Intrinsically Disordered Region

**Figure 5:**
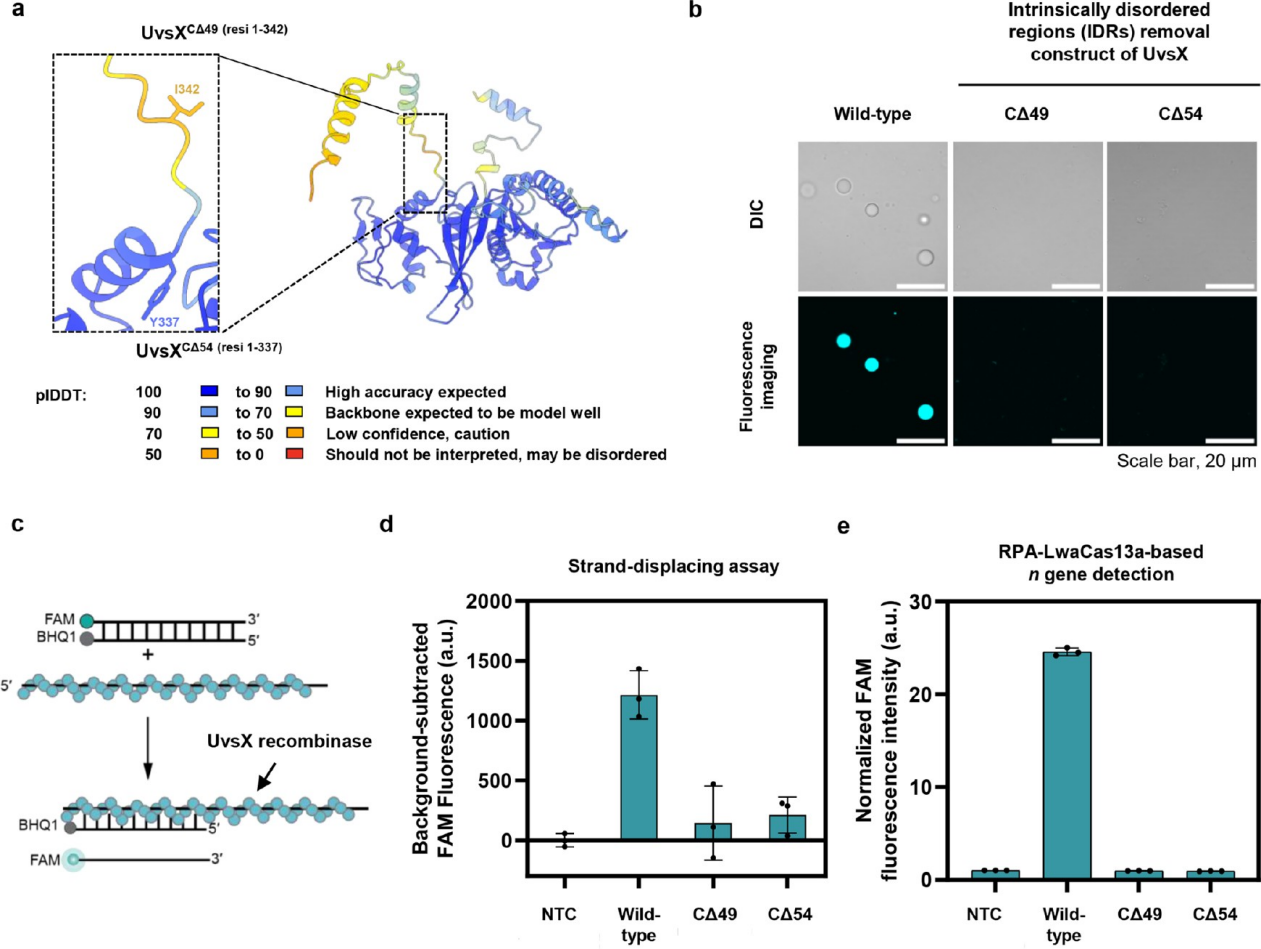
Intrinsically disordered C-terminus of UvsX mediates its phase separation. (a) AlphaFold prediction of T4 UvsX structure. Per-residue model confidence scores (pLDDT) were pseudocolored onto the predicted structure. Low-pLDDT, orange-colored regions are likely intrinsically disordered. (b) Confocal imaging of wild-type UvsX vs C-terminal truncated UvsX mutants UvsX^CΔ49^ and UvsX^CΔ54^. Representative images were obtained with 4.7 μM UvsX and 0.2 μM AZ405-labeled UvsX in an RPA buffer (50 mM Tris-HCl pH 7.5, 100 mM KOAc, 14 mM Mg(OAc)_2_, 2 mM DTT, 5% (w/v) PEG20000). Images are representative of three replicates. Scale bar, 20 μm. Additional fields of view are in Figure S11. (c) Schematic diagram of the strand displacement assay. UvsX assembles on unlabeled ssDNA to form a nucleoprotein filament. UvsX searches for homologous sequences to ssDNA, facilitating DNA strand exchange into FAM-BHQ1-dsDNA. Upon release of the noncomplementary strand (a FAM-labeled oligo), fluorescence emission is observed. (d) Strand exchange reactions driven by UvsX and its variants. FAM fluorescence was subtracted against intensities obtained from the UvsX-free condition (buffer) at 60 min. Data are presented with three independent replicates. Raw data are in Figure S13a. (e) RPA followed by LwaCas13a-based *n* gene detection were performed with 10,000 copies of pUC57–2019-nCoV-N plasmid as template. Three replicates of the amplification/detection reactions were performed. FAM fluorescence at 60 min were normalized against intensities obtained from the no template control. Raw data are shown in Figure S13b.

Intrinsically disordered regions (IDRs) are often essential for phase-separating proteins as they contribute to multivalency by mediating weak interactions between components.^35,36^ AlphaFold structural prediction^37^ and FlDPnn Web server^38^ indicated that *T4* UvsX contains IDRs at its C-terminus (Figures 5a, S10 and S11a). This C-terminal region from residue 338 onward exhibited low pLDDT values according to the AlphaFold prediction, highlighting residue-wise disorder within the region (Figure 5a) and consistent with the lack of electron densities from the region in the reported crystal structure of UvsX.^14^ We prepared two UvsX mutants with truncated C-termini: UvsX^CΔ49^ (truncation from amino acid position 343 onward) and UvsX^CΔ54^ (truncation from position 338 onward). Neither mutant could form condensates (Figures 5b and S11b), indicating that the C-terminal IDRs are critical for phase separation of UvsX. We then evaluated the activities of truncated UvsX mutants using RPA followed by CRISPR-based detection, as well as an assay to directly monitor strand displacement catalyzed by UvsX. In the latter, we used a Förster resonance energy transfer (FRET)-based real-time strand exchange assay similar to previous studies.^39^ In short, FAM and its quencher BHQ1 were introduced into opposing strands of the DNA duplex. FAM fluorescence would be effectively quenched by BHQ1 via FRET when the duplex is intact. Strand exchange and subsequent ssDNA release catalyzed by UvsX would separate FAM from BHQ1, relieving quenching and causing an increase in FAM fluorescence signal (Figure 5c; assay validation in Figure S12). The removal of the disordered region at the C-terminus of UvsX dramatically impeded its function in such a strand-displacing activity assay (Figures 5d and S13a), as well as in RPA reactions (Figures 5e and S13).

The C-terminus of UvsX shares a similar feature to that of RecA in that it contains several acidic residues which modulate dynamic binding to DNA.^40^ Here, we showed its additional function as an IDR, which modulates phase separation of UvsX. Truncation of the C-terminus of UvsX, therefore, affects both its DNA strand exchange activity and phase separation, leading to cumulative negative effects on nucleic acid amplification in RPA, a process which requires both enzymatic and physical phase-separating capabilities of UvsX.

### Droplet Size is Not the Major Determinant of Different RPA Activities Observed Across UvsX Concentrations

**Figure 6:**
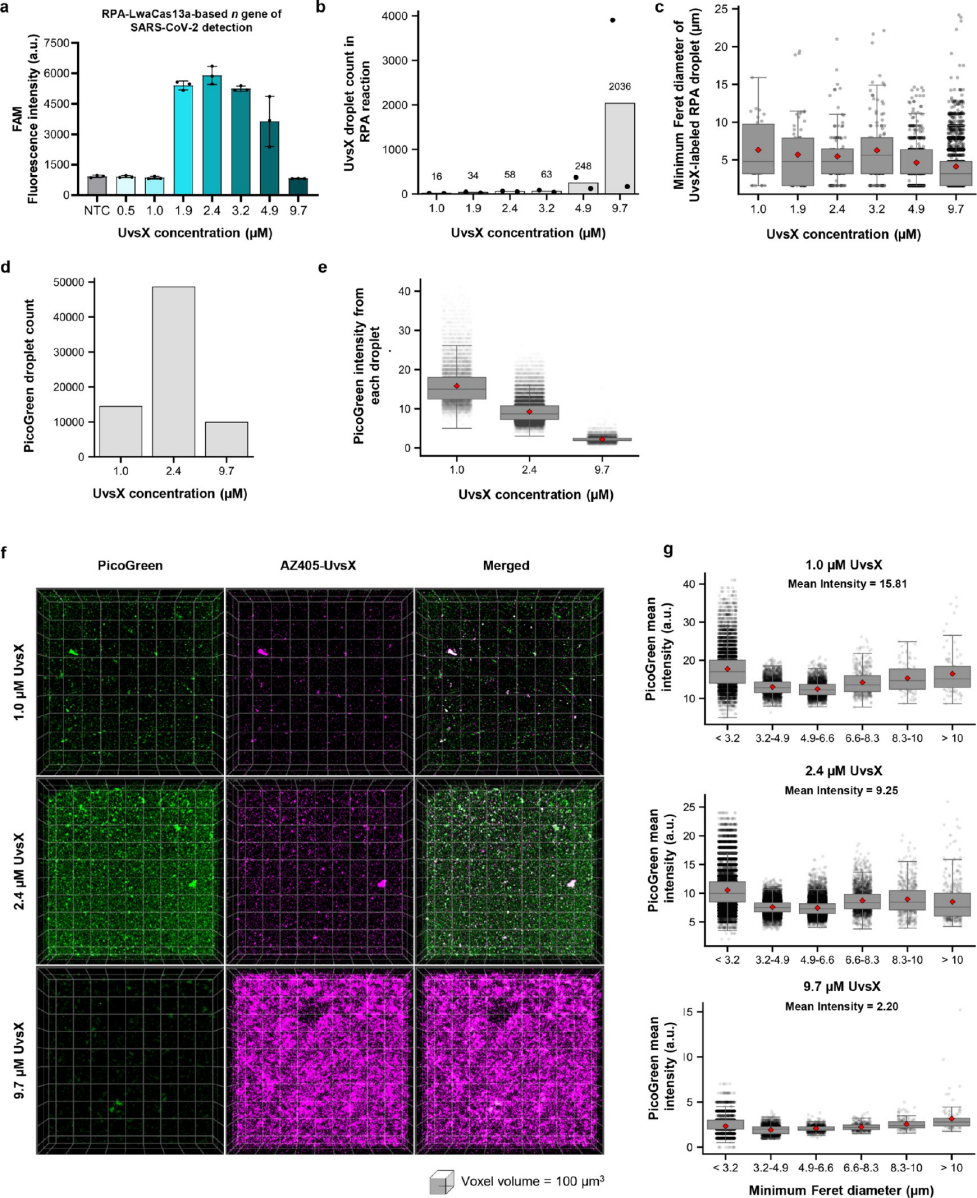
Characterizations of RPA droplet number and size distributions across UvsX concentrations. (a) RPA efficiency at different UvsX concentrations. RPA was performed using 10,000 copies of pUC57–2019-nCoV-N plasmid as input, followed by LwaCas13a-based *n* gene detection. NTC, negative control with RNase-free water as input. NTC background-subtracted FAM fluorescence intensities generated from Cas13a reactions after 60 min are shown. Reactions at each UvsX concentration were performed in triplicate. (b,c) Number and distribution of minimum Feret diameter of UvsX-AZ405 labeled droplets under different concentrations of UvsX in RPA reactions at 30 min. Representative maximum intensity projection images of AZ405-labeled droplets are shown in Figure S15. (d,e) Number and distribution of mean PicoGreen intensities of PicoGreen-labeled droplets under different concentrations of UvsX in RPA reactions at 15 min. Representative maximum intensity projection images of PicoGreen-stained droplets are shown in Figure S17b. (f) Two-color imaging of AZ405-labeled UvsX and PicoGreen in RPA droplets. RPA reactions with labeled UvsX were performed using the *n* gene plasmid as the DNA template with varying UvsX concentrations (1.0 μM, 2.4 μM, and 9.7 μM) and incubated for 30 min, then stained with PicoGreen. *Z*-series images were collected from the bottom to the top of the well, and representative 3D images of PicoGreen- (green) and AZ405-labeled (magenta) RPA droplets are shown; overlapped regions of the two labels appear as white. (g) Distribution of mean PicoGreen intensities of PicoGreen-labeled droplets separated by droplet size (minimum Feret diameter) under different concentrations of UvsX in RPA reactions at 15 min, from the same underlying data as d-e and Figure S17b.

Our previous effort to formulate activity-optimized RPA revealed a narrow range of concentrations of UvsX (2–3 μM) optimal for DNA amplification.^9^ Higher (up to 9.7 μM) and lower (down to 500 nM) concentrations of UvsX than the optimal range resulted in much-decreased DNA amplification efficiencies (Figure 6a). To understand why this specific concentration range produced the best results, we examined the properties of droplets formed at various UvsX concentrations, focusing on their count, size distribution, and relationship to the reaction efficiency. In particular, we initially thought the droplet size could play a key role in determining RPA efficiency: larger droplets could reduce RPA reaction efficiency by creating “dead” volumes or hindering substrate diffusion, similar to size-dependent reactivity trends observed with nanoparticle catalysts.^41^

We first imaged fluorescently tagged UvsX droplets that had sedimented to the well bottom using confocal imaging and found there was a UvsX concentration-dependent increase in overall droplet size (Figure S14a,b). However, we later found that size estimation of droplets based on measurements at the well bottom was misleading, as higher proportions of droplets were accumulating at the bottom compared to the rest of the well volume (Figure 2d), and contact-dependent droplet fusion, resulting in larger droplets, could be easily promoted at the well bottom due to higher droplet concentrations.

We then switched to characterizing the RPA droplet count and size distribution in whole reaction volumes through 3D imaging. We observed a greater number of droplets at higher UvsX concentrations (Figures 6b and S15), but roughly the same median RPA droplet diameter (∼4–6 μm) across 1.0–9.7 μM UvsX used in RPA (Figure 6c). At 9.7 μM UvsX, where RPA activity is largely suppressed, we saw both higher counts of smaller (diameter ≤10 μm, Figure S16) and larger droplets (diameter >10 μm, Figure S16) than at other UvsX concentrations.

To test whether RPA droplets of different sizes could differentially contribute to RPA activity, we performed volumetric imaging to analyze the RPA products via PicoGreen fluorescence with UvsX supplied at 1.0 μM, 2.4 μM, and 9.7 μM (Figure 6d,e; negative controls at Figure S17). At 1.0 μM UvsX, we detected few PicoGreen-positive RPA droplets (Figure 6d,e), consistent with the CRISPR-based detection result of low RPA efficiency at this UvsX concentration, but the mean PicoGreen intensity per droplet under this condition was the highest (mean 15.81 au). At 2.4 μM UvsX, the reaction produced a 3.3-fold higher count of PicoGreen-positive droplets than at 1.0 μM UvsX while retaining high PicoGreen intensity per droplet (mean 9.25 au), cumulatively resulting in the highest CRISPR-based detection signal observed at this condition. At 9.7 μM UvsX, few PicoGreen-positive droplets were observed, and the mean PicoGreen intensity per droplet (mean 2.20 au) was the lowest among the three UvsX concentrations tested. Two-color volumetric imaging of RPA droplets with PicoGreen and AZ405-labeled UvsX showed that PicoGreen costained well with fluorescent UvsX signals in droplets, particularly at lower UvsX concentrations (Figure 6f).

Under each UvsX concentration condition, we analyzed mean PicoGreen intensities per droplet for RPA droplets of varying sizes present in the reactions (Figure 6g). We observed similar mean PicoGreen intensities per droplet at a given UvsX concentration, regardless of the droplet size. Therefore, the droplet size is not the major determinant of different RPA activities observed under different UvsX concentrations. One possibility is that the excessive droplet formation at 9.7 μM by UvsX disrupts the stoichiometric balance of components in the RPA reaction. The system relies on the coordinated activity of UvsX, UvsY, Gp32, and *Bsu* Pol. At elevated UvsX levels, the disproportionate formation of droplets may result in an uneven distribution of these components, leading to inefficient recruitment into individual droplets and failure to form functional enzymatic complexes. At 2–3 μM, there may be an optimal balance between a sufficiently high RPA droplet count and optimal distributions of RPA components within droplets, resulting in optimal reaction performance.

While higher UvsX concentrations lead to faster consumption of ATP, we verified that the remaining amount of ATP (4–5 mM) after a 60-min RPA reaction with 4.9 μM UvsX is still well above the *K*_m_ for ATP by UvsX (*K*_m_ = 0.15–0.39 mM for the ADP production pathway^18^ needed to drive branch migration) and could readily catalyze RPA (Figure S18), suggesting ATP is not a limiting factor for RPA progression even at higher UvsX concentrations.

### Specific Organizations within RPA Multiphase Condensates Likely Contribute to the Reaction Efficiency

**Figure 7:**
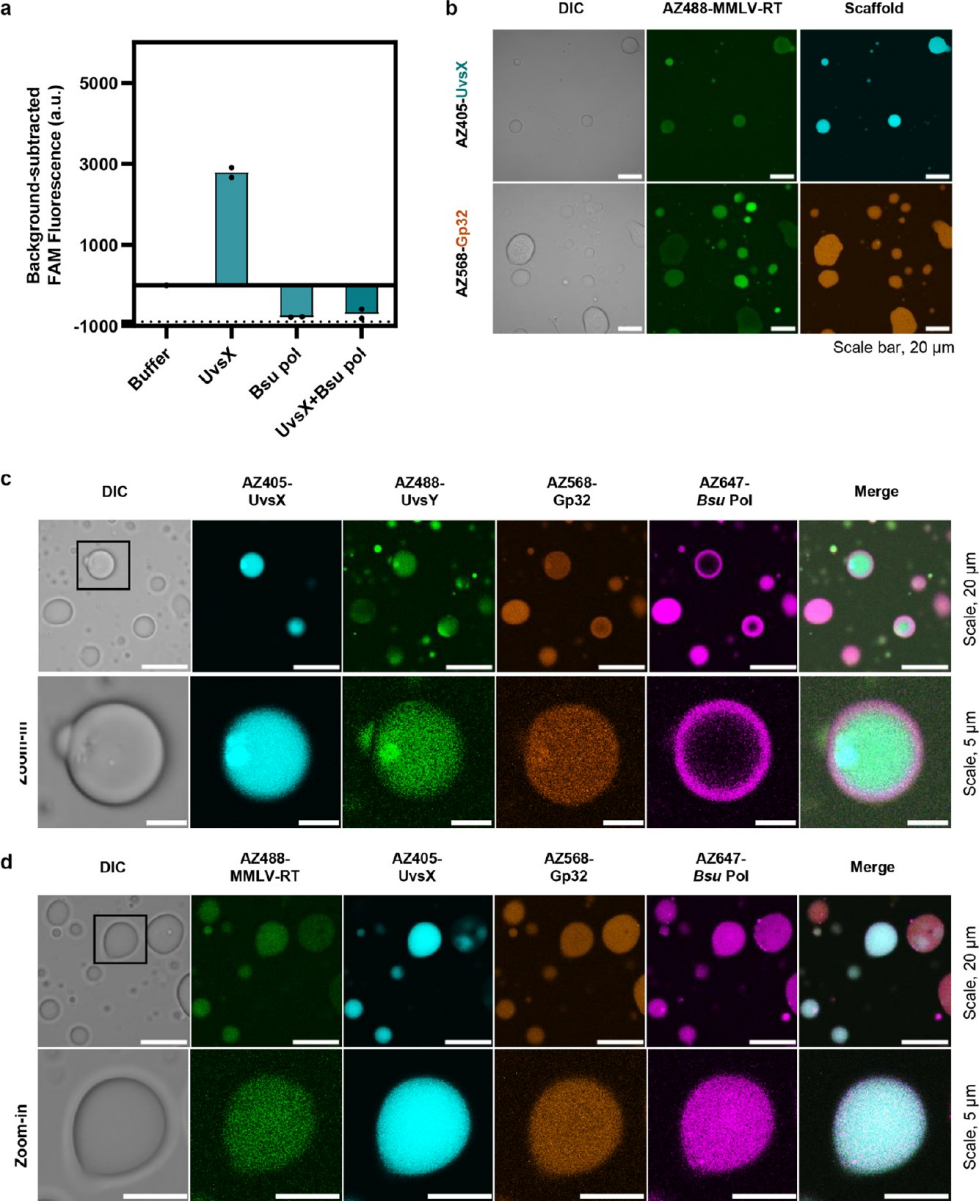
RPA vs reverse-transcription RPA condensates. (a) Strand-displacing activity of UvsX was inhibited in the presence of *Bsu* Pol. The final protein concentrations in all reactions were 1.3 mg/mL, matching the total protein concentration used in the RPA amplification assay. In the UvsX + *Bsu* Pol condition, the total protein concentration was 1.3 mg/mL, equating to 0.65 mg/mL for each protein. FAM fluorescence values were normalized by subtracting intensities obtained from the UvsX-free condition (buffer). Data from two replicates are shown. Raw data are shown inFigure S19. (b) Confocal images of reverse transcriptase (RT)-UvsX and RT-Gp32 condensates. Concentrations of the mixed proteins were as follows: 3.3 μM UvsX with 0.2 μM AZ405-labeled UvsX; 26 μM Gp32 with 0.2 μM AZ568-labeled Gp32; and 0.1 μM MMLV-RT with 0.3 μM AZ488-labeled MMLV-RT. The mixtures were prepared in RPA buffer (50 mM Tris-HCl pH 7.5, 100 mM KCl, 14 mM MgOAc, 2 mM DTT, 5% (w/v) PEG20000). Scale bars, 20 μm. Additional views are in Figure S21a-–d. (c) Confocal images of RPA multiphase condensates. Concentrations of the mixed proteins mimicked those found in the RPA amplification reaction: 3.3 μM UvsX with 0.2 μM AZ405-labeled UvsX; 3.3 μM UvsY with 0.2 μM AZ488-labeled UvsY; 26 μM Gp32 with 0.2 μM AZ568-labeled Gp32; and 1.8 μM *Bsu* Pol with 0.2 μM AZ647-labeled *Bsu* Pol. The mixture was prepared in RPA buffer. Zoom-ins of the boxed droplets on the top panel are shown. Images are representative of three replicates. Additional views are in Figure S23. (d) Confocal images of RT-RPA condensates. Concentrations of the mixed proteins were as follows: 3.3 μM UvsX with 0.2 μM AZ405-labeled UvsX; 3.3 μM UvsY, 26 μM Gp32 with 0.2 μM AZ568-labeled Gp32; 1.8 μM *Bsu* Pol with 0.2 μM AZ647-labeled *Bsu* Pol; and 0.1 μM MMLV-RT with 0.3 μM AZ488-labeled MMLV-RT. The mixture was prepared in RPA buffer. Zoom-ins of the boxed droplets on the top panel are shown. Images are representative of three replicates. Additional views are in Figure S21e.

Specific protein organizations within the multiphase condensates could also confer an advantage to the RPA activity. RPA fundamentally requires two key enzymatic activities—strand exchange mediated by the recombinase, and DNA synthesis mediated by the polymerase—yet we observed the two enzymes responsible for both activities (UvsX and *Bsu* Pol) to be spatially segregated in the biphasic condensates. To investigate the potential incompatibility of the two enzymes, we measured the strand-displacing activity of UvsX in the presence of *Bsu* Pol, without phase separation (by omitting the crowding agent PEG20000 from the reactions), and found UvsX activity to be completely suppressed by *Bsu* Pol (Figures 7a and S19). While the mechanism for such activity suppression is unclear, the suppression was independent of the presence of dNTPs, suggesting that the effect was not contingent on *Bsu* Pol-mediated DNA synthesis. The *E. coli* homologue of UvsX, RecA, is known to maintain its activity in the presence of *Bsu* Pol^42^ (and in the absence of dNTPs), so the suppression by *Bsu* Pol observed may be specific to *T4* UvsX.

In contrast to the in-solution activity incompatibility of UvsX and *Bsu* Pol, we observed that UvsX can catalyze strand exchange in the presence of *Bsu* Pol when they are phase-separated within condensates. Here, we monitored the strand-displacing activity of UvsX using the aforementioned FAM/BHQ1-labeled DNA duplex within condensates; similar to before, strand exchange mediated by UvsX would separate FAM-labeled ssDNA from its BHQ1-labeled opposing strand, resulting in FAM fluorescence visible in droplets. We first confirmed that the generation of FAM fluorescence in condensates is dependent on the presence of UvsX, also in condensates and labeled with AZ405, and the invading unlabeled ssDNA (Figure S20a–d). We then assembled the full RPA reaction using fluorescently labeled UvsX, Gp32, and *Bsu* Pol (and unlabeled UvsY) and observed FAM signal generation within RPA droplets (Figure S20e). The FAM signal largely colocalized with UvsX in droplets, whereas *Bsu* Pol formed concentrated puncta at the droplet periphery, suggesting that spatial segregation of UvsX and *Bsu* Pol within the RPA droplets may be crucial for UvsX activity.

Reverse transcription-RPA to amplify target sequences derived from RNA is known to proceed at lower efficiencies than regular RPA,^24,43^ partly due to the generation of RNA-DNA hybrids, which obstruct RPA.^43^ The addition of RNase H to resolve RNA-DNA hybrids can boost RT-RPA efficiency, but not to the same level as regular RPA with DNA as substrates.^43^ We wondered if the interplay between the reverse transcriptase (RT) enzyme and other RPA proteins—in the context of condensates—could additionally affect the reaction efficiency. We expressed, purified, and fluorescently labeled Moloney Murine Leukemia Virus reverse transcriptase (MMLV-RT) harboring D525G/E563Q/D584N mutations in the ribonuclease H (RNase H) domain; this RT variant was chosen for its enhanced reverse transcription efficiency due to minimized degradation of the RNA template. Notably, RT itself does not undergo phase separation but could be recruited into condensates when combined with scaffold proteins UvsX or Gp32 (Figures 7b and S21). UvsX mutants lacking the intrinsically disordered C-terminal regions were not able to form condensates, nor recruit RT (Figure S23). RT recruitment into condensates by Gp32 differentiated it from other client proteins of RPA (UvsY and *Bsu* Pol), which could be recruited mainly by UvsX.

We next observed the formation of condensates upon mixing RPA proteins together in the presence and absence of the RT enzyme (Figure 7c,d). In the absence of MMLV-RT, the four protein components of RPA showed spatial arrangements consistent with their behavior in pairwise imaging experiments (Figure 4). UvsY largely colocalized with UvsX at the core of condensates. Gp32 showed stronger concentrations at the shell layer but was also present at the core. *Bsu* Pol showed heterogeneous localization patterns but could form distinct shells and concentrate into puncta at the droplet edge (Figures 7c and S23). In contrast, *Bsu* Pol’s movement toward the droplet edge within the RPA mixture was completely absent when MMLV-RT was present (Figures 7d and S21e). Fully miscible UvsX and *Bsu* Pol within RT-RPA droplets and the likely resulting effect of reduced UvsX activity due to inhibition by *Bsu* Pol may contribute to the worse efficiency of RT-RPA when compared to RPA. Beyond spatial segregation of protein components, which could contribute to their optimal activities within condensates, differences in local protein concentrations within condensates, which we have not characterized, may also affect the reaction efficiencies in the presence/absence of RT in the RPA reaction.

### UvsX^D274A^ Has Distinct Phase-Separation Properties and Further Enhances RPA

**Figure 8:**
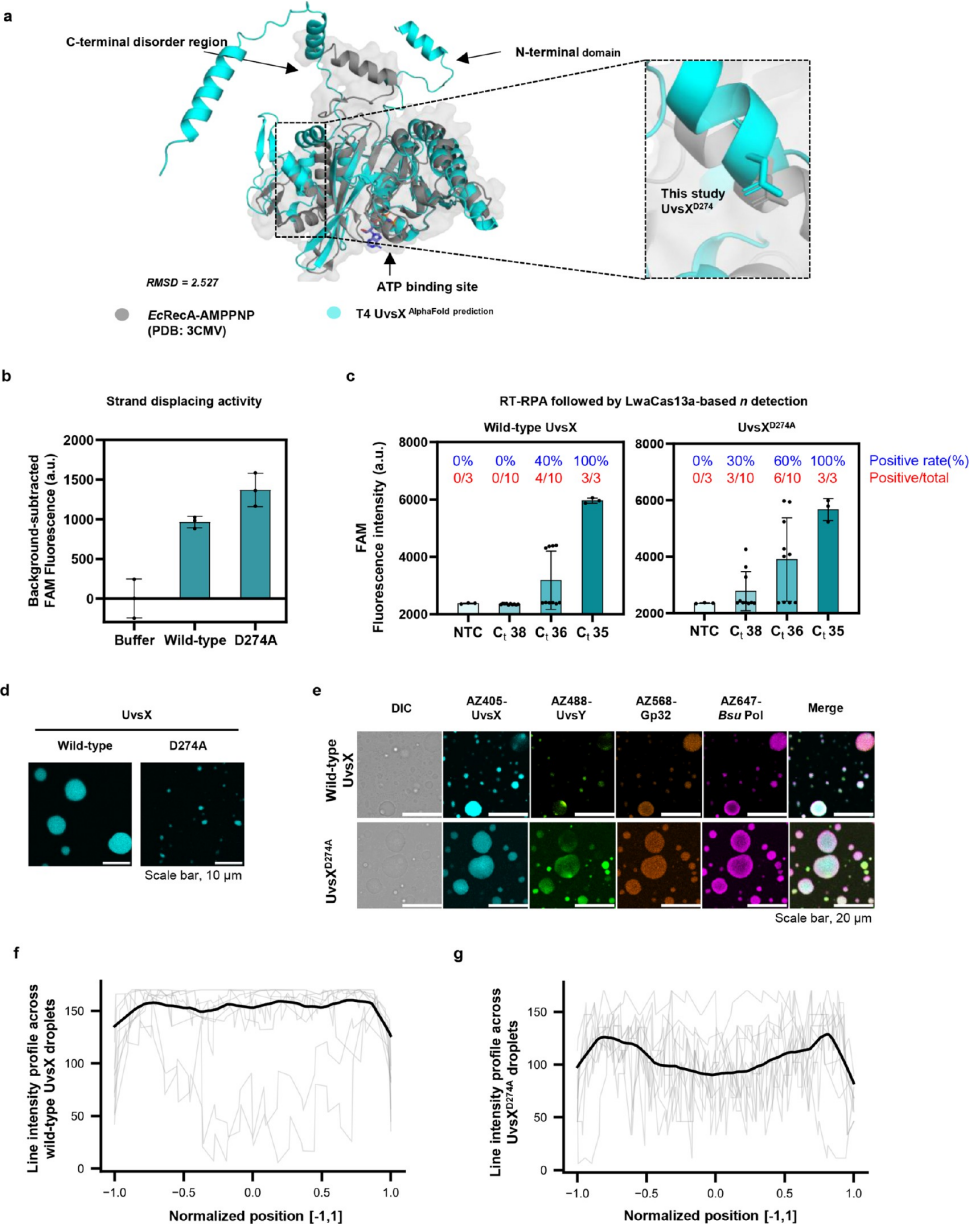
UvsX^D274A^ has distinct phase-separation properties and improves RPA. (a) Structural comparison of *E. coli* RecA (PDB: 3CMV) and T4 UvsX (AlphaFold prediction). Key regions crucial for specific functions are highlighted with arrows. D274 of *T4* UvsX and the analogous D276 of RecA are highlighted. (b) Strand-displacing activity of wild-type UvsX and UvsX^D274A^ variant. FAM fluorescence was subtracted against intensities obtained from the UvsX-free condition (buffer) at 60 min. Data from three independent replicates are shown. Raw data are in Figure S26a. Error bars, ± s.d. (c) Activity comparison of wild-type UvsX (left) to UvsX^D274A^ (right) in the RT-RPA for amplification of the *n* gene of SARS-CoV-2. RT-RPA followed by LwaCas13a-based detection was performed with serially diluted SARS-CoV-2 RNA, whose *C*_t_ values were determined using a Luna one-step RT-qPCR assay targeting the *n* gene of SARS-CoV-2. Ten replicates of the amplification/detection reactions were performed for SARS-CoV-2 RNA dilution at *C*_t_ 36 and 38, while three replicates were performed for *C*_t_ 35 and negative control (NTC, with RNase-free water as reaction input). NTC background-subtracted FAM fluorescence intensities at 120 min are shown. Raw kinetic traces of FAM fluorescence generation are shown in Figure S27. (d) Confocal imaging and sizes of UvsX^D274A^ in comparison to wild-type UvsX. Representative images were obtained in phase-separation experiment in samples containing 3.8 μM UvsX or UvsX^D274A^ and 0.3 μM AZ405-labeled UvsX or AZ405-labeled UvsX^D274A^. Additional fields of view are in Figure S28. (e) Confocal imaging of RPA condensates with UvsX^D274A^ in comparison to RPA condensates with wild-type UvsX. Concentrations of mixed proteins mirrored those present in the RPA reaction, as follows: 3.3 μM UvsX with 0.2 μM AZ405-labeled UvsX^D274A^; 3.3 μM UvsY with 0.2 μM AZ488-labeled UvsY; 26 μM Gp32 with 0.2 μM AZ568-labeled Gp32; and 1.8 μM *Bsu* Pol with 0.2 μM AZ647-labeled *Bsu* Pol. The mixture was prepared in an RPA buffer containing 50 mM Tris-HCl pH 7.5, 100 mM KOAc, 14 mM Mg(OAc)_2_, 2 mM DTT, and 5% (w/v) PEG20000. Scale bars, 20 μm. Additional fields of view are in Figure S29. (f–g), Line-profile analysis of *Bsu* Pol fluorescence intensities in RPA condensates with UvsX^D274A^ in comparison to UvsX. Lines were drawn across nine droplets from UvsX^D274A^ and eight droplets from wild-type UvsX condition, then position values were normalized to a scale of [−1,1]. Within the normalized position range, intensity profiles from droplets were averaged to generate representative curves, which were smoothed using locally weighted scatterplot smoothing (LOWESS).

With the insight that RPA functions as a multiphase condensate whose formation is controlled by the UvsX recombinase, we wondered if RPA could be improved through modulation of UvsX activity and phase-separation propensity. As a starting point, we took a conservative engineering approach whereby we transplanted point mutations of *E. coli* and *Deinococcus radiodurans* RecA—both of which are close sequence and structural homologues of T4 UvsX (Figures 8a and S24)—known to improve homologous recombination, to analogous amino acid positions in UvsX. For example, *E. coli* RecA^D112R^ exhibits an enhanced capacity to load onto SSB-coated single-stranded DNA (ssDNA) and produce high recombination frequencies.^44^ The radiation-resistant bacterium *Deinococcus radiodurans* possesses a distinctive glycine at position 82, in contrast with the conserved serine found in many organisms. This substitution in DrRecA’s ATP binding site enhances dATP hydrolysis and SSB displacement from ssDNA compared to ATP.^45^ We hypothesized that the substitution of Ser64 of UvsX with glycine might increase the rate of dATP hydrolysis and SSB displacement from ssDNA and improve the RPA reaction. Moreover, an *E. coli* strain was subjected to strain evolution to generate an infrared-resistant strain variant.^46,47^ The resulting mutations to RecA (D276A and D276N) exhibit a faster rate of filament nucleation on DNA, more effectively facilitate strand exchange, and reduce the inhibitory action of ADP.^48^ We thus thought that an analogous mutation to UvsX (at position 274) may also have beneficial effects. Importantly, all three point mutations we selected (S64G, D106R, and D274A) are radical replacements, with an exchange of amino acid residues with different physicochemical properties. While the mutations are not at the C-terminal IDR region of UvsX, they could still affect the micropolarity of UvsX, modulate its multivalent interactions and phase-separation behavior, and ultimately, RPA efficiency.

After expression and purification (Figure S25), we first measured the strand-displacing activities of the UvsX mutants and found them to largely maintain good levels of activity when compared to wild-type activity (Figure S26a). We further assessed the UvsX mutants in an RPA assay using DNA as input. Initial screening reactions used concentrated DNA input, followed by subsequent rounds of gradually diluted DNA input and shorter reaction times. Ultimately, the UvsX^D274A^ mutant emerged as the top candidate, showcasing excellent performance in the RPA reaction, particularly upon using lower copy numbers of DNA input (Figure S26b), higher strand-displacing activity than the wild-type enzyme (Figure 8b), and higher sensitivity in amplifying genes from RNA, judging by higher positive rates (60% for *C*_t_ 36 sample; 30% for *C*_t_ 38 sample) of amplification of SARS-CoV-2 RNA with higher cycle threshold values (and therefore lower RNA input) than the wild-type enzyme (40% for *C*_t_ 36 sample; 0% for *C*_t_ 38 sample, Figures 8c and S27).

We next investigated the characteristics of UvsX^D274A^ droplet formation and its potential relationship to its function in nucleic acid amplification. We observed that UvsX^D274A^ readily formed more compact condensates than those formed by wild-type UvsX (Figures 8d and S28). The more compact droplets may be consistent with the increase in microhydrophobicity of UvsX^D274A^ due to the charged aspartate-to-uncharged alanine mutation and the resulting higher interfacial free-energy densities. The change in micropolarity of UvsX^D274A^ was further supported when we examined its localization within the multiphase condensates of RPA. UvsX^D274A^ still formed the core layer of the RPA condensates, but its core–shell separation from *Bsu* Pol became more consistent, in contrast to the heterogeneous core–shell separation observed with wild-type UvsX (Figures 8e–g S29). UvsX^D274A^ thus may improve the RPA reaction beyond the wild-type enzyme through related mechanisms relevant to phase separation: enhanced microhydrophobicity of the core layer, which promotes better electrostatic interactions between UvsX^D274A^ and nucleic acids; and more uniform segregation of UvsX^D274A^ and *Bsu* Pol in the core–shell arrangement within condensates, which reduces inhibition of UvsX^D274A^ by *Bsu* Pol while enabling efficient substrate exchange between the two enzymes.

## Discussion

In summary, we discovered that a widely used recombinase-mediated nucleic acid amplification reaction, RPA, functions as multiphase condensates, with UvsX recombinase—long known to be the key enzyme for homology search and strand invasion within RPA—acting as a dynamic scaffold protein and a spatial organizer of other proteins within condensates. We identified the intrinsically disordered tail of UvsX as crucial to its phase-separation propensity and investigated the spatial organization of RPA components, such as UvsX–*Bsu* Pol, that likely play key roles in optimizing RPA activity. We created volumetric imaging assays to observe RPA condensates and track the progression of the reaction across entire volumes, and explored how macroscopic factors like droplet size distribution and count might impact the overall efficiency of the reaction, as well as an assay to measure recombinase activity directly in droplets.

Nevertheless, we recognize several limitations with our current methodologies, which in turn motivate our future method developments. Volumetric imaging at low magnification can image μL-reaction volumes and visualize free-floating droplets, but at too low a spatial resolution to pinpoint detailed organizations (such as the core–shell structure) within droplets. Higher-magnification imaging of sedimented droplets at the coverslip-sample interface can provide more detailed spatial information down to the diffraction limit with conventional fluorescence imaging, but the physicochemical properties of sedimented droplets are likely not the same as free-floating ones. Due to assay limitations, we were not able to investigate combined macromicroscopic properties, such as organizations of RPA protein components in whole reaction volumes, which would have provided critical insight into how condensate microstructures affect nucleic acid amplification of RPA.

The insight that phase separation of RPA is critical to the reaction efficiency will be useful for further optimizations of the reaction, as we have already demonstrated with UvsX concentration-dependent effects on the reaction efficiency, UvsX engineering, as well as the investigation into the lower efficiency of reverse transcription-RPA. Local microenvironments within biomolecular condensates are shown to alter enzymatic activity through a variety of mechanisms, such as an increase in local enzyme concentration within condensates^49^ and distinct electrostatic properties^50^ and hydrophobicity.^51^ Physical properties like viscosity^52^ and pH^53^ are further shown to be modulated within biomolecular condensates, all of which could affect enzyme activity through changing the availability of substrates and enzyme conformations. While we are just beginning to understand how the chemical and physical properties of RPA condensates contribute to its efficiency and function, we believe that, at a minimum, reaction optimizations while keeping its phase-separation propensity in mind are crucial to maintain RPA efficiency in different biological fluids and sample types.

Ultimately, dissecting the contributions of different physicochemical parameters to the overall amplification efficiency of RPA could pave the way for much-improved point-of-care genotyping and diagnostic applications of the reaction. Recent developments in diagnostic technologies using CRISPR and isothermal amplifications are poised to make an impact on accurate genetic and disease testing at the point of care or at home. Keys to improving these tests for potential broader use, according to the REASSURED guideline^54^ are to ensure the robustness of tests while maintaining test affordability. Protein engineering to improve the efficiencies of these tests at the molecular level, as we have done with UvsX, could complement other improvement efforts such as device design, simplifying specimen collection, and enabling real-time connectivity.^11,55^

Beyond diagnostics, we believe that RPA may serve as a good *in vitro* model system for multiphase condensate studies, which currently rely on small polypeptides with defined physicochemical properties for the investigation of structural organizations within condensates. The nucleolus represents a well-studied, biologically relevant multiphase condensate system^56^ which can be reconstituted *in vitro* (such as the nucleophosmin-fibrillarin condensates,^57^ but was also investigated mainly from the structural organization standpoint. The RPA condensate system is distinct from other model systems in that correlation between catalytic improvements (to the recombinase activity or to the overall nucleic acid amplification) and organization within condensates can be made. This distinction should enable systematic engineering of the reaction components of RPA and ultimately enable us to determine how molecular-level changes to the components can affect their organizations and catalysis within condensates.

## Data Availability

All datasets generated and analyzed here are available from the corresponding author upon reasonable request.

## Supporting Information Available

Additional experimental details, materials, and methods, including primer and oligonucleotide sequences used in the study, protein expression and purification procedures, RPA conditions, UvsX activity assay conditions, and imaging protocols and analyses. The file also contains Supplementary Figures 1–29 (PDF)
