# Supplementary material for: Recombinase-Controlled Multiphase Condensates Accelerate Nucleic Acid Amplification and CRISPR-Based Diagnostics
Source: J Am Chem Soc. 2025 Feb 13;147(12):10088–103. doi: 10.1021/jacs.4c11893 (PMC11951158; doi:10.1021/jacs.4c11893)
Supplement: Supplementary file 1 — ja4c11893_si_001.pdf [file ja4c11893_si_001.pdf]

Supporting Information

Recombinase-controlled multiphase condensates accelerate nucleic acid amplification and CRISPR-based diagnostics

Aimorn Homchan[1†], Maturada Patchsung[1†], Pheerawat Chantanakool[1†],Thanakrit Wongsatit[1†], Warunya Onchan[1], Duangkamon Muengsaen[1], Thana Thaweeskulchai[1], Martin Tandean[1], Theeradon Sakpetch[1], Surased Suraritdechachai[1], Kanokpol Aphicho[1], Chuthamat Panchai[1], Siraphob Taiwan[1], Navin Horthongkham[2], Taweesak Sudyoadsuk[3], Aleks Reinhardt[4], Chayasith Uttamapinant[1*]

[1]School of Biomolecular Science and Engineering, Vidyasirimedhi Institute of Science and Technology (VISTEC), Rayong 21210, Thailand
[2]Department of Microbiology, Faculty of Medicine Siriraj Hospital, Mahidol University, Bangkok 10700, Thailand
[3]Frontier Research Center, Vidyasirimedhi Institute of Science and Technology (VISTEC), Rayong 21210, Thailand
[4]Yusuf Hamied Department of Chemistry, University of Cambridge, Cambridge CB2 1EW, United Kingdom

*Correspondence: chayasith.u@vistec.ac.th
†These authors contributed equally to this work

This file includes:

Supplementary Fig. 1 to 29 and Materials and Methods

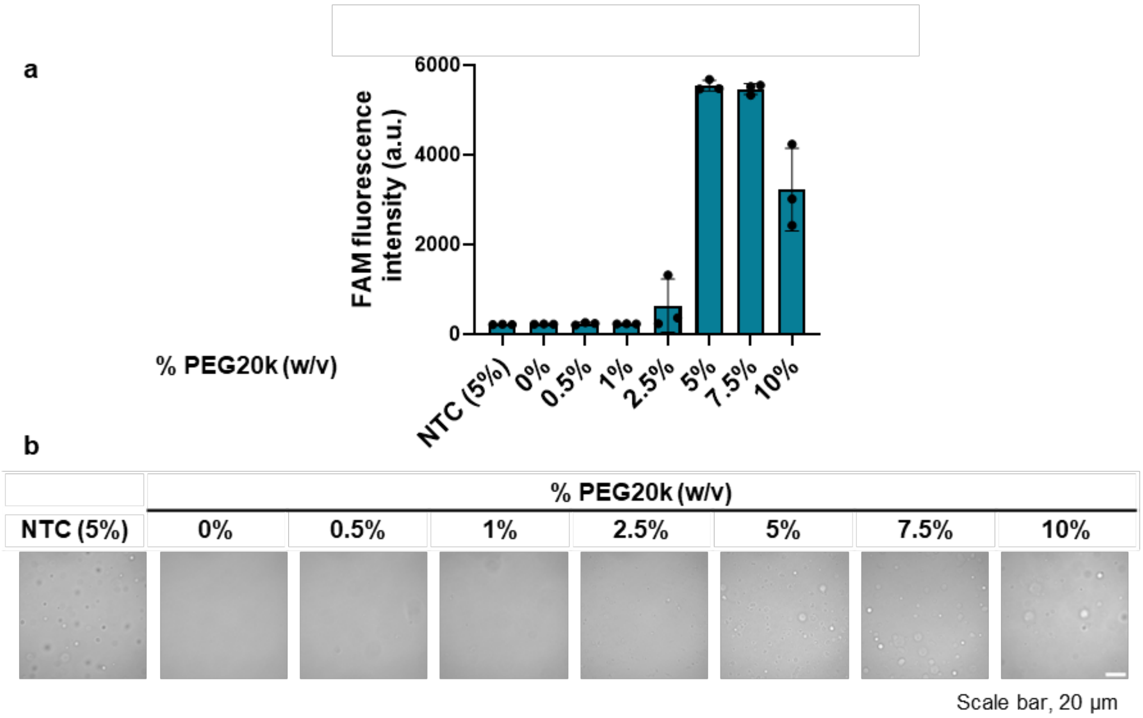

Supplementary Fig. 1. **a, Locally produced RPA required the crowding agent PEG20000.** RPA reactions were carried out with different concentrations of PEG20000, using the pUC57-2019-nCoV-N plasmid as a template (10,000 copies). a, The FAM fluorescence intensity resulting from LwaCas13a-based *n* gene detection. **b, Brightfield images of the RPA reactions at different %PEG20000.** Images were taken at the endpoint of the reactions. Scale bar, 20 µm.

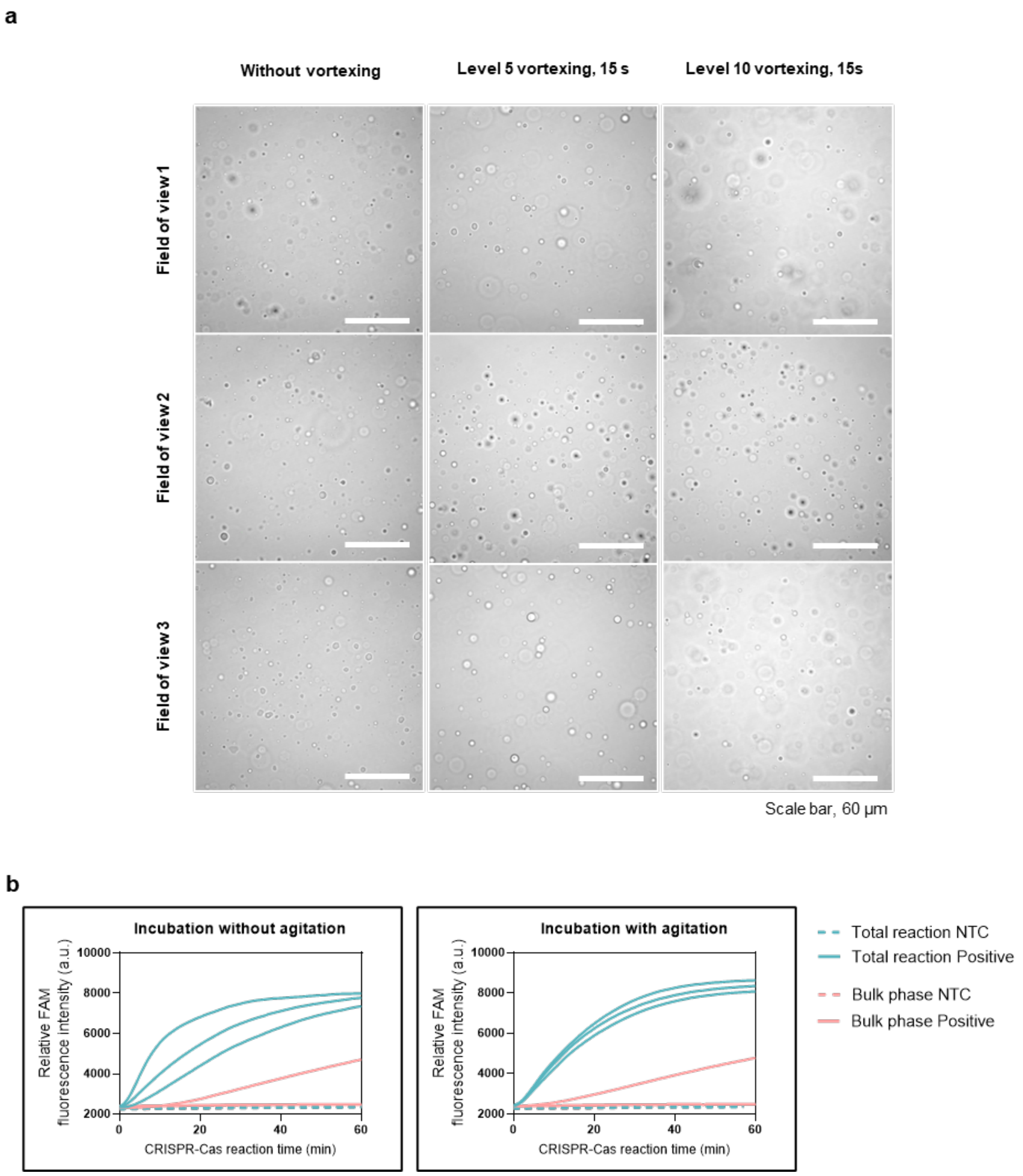

Supplementary Fig. 2. **RPA droplet persistence and RPA efficiency in the presence of mechanical agitation**

a, Brightfield images of RPA droplets before and after two different settings of vortexing (level 10 is maximal speed), showing persistence of the droplets even after vortexing. **b**, Following the preparation of the RPA reaction, aliquots were taken to represent the total reaction. The remaining RPA reactions were centrifuged to separate the bulk and droplet phases. RPA amplification was performed using 10,000 copies of the pUC57-2019-nCoV-N plasmid as the DNA template. Negative control reactions were conducted using RNase-free water in place of the DNA input. The total RPA reaction and the bulk phase solution were incubated under two conditions: with and without agitation. For the agitation condition, a ThermoMixer (Eppendorf) was used to mix and incubate the samples simultaneously. The LwaCas13a-based detection of the *n* gene of SARS-CoV-2 was performed on the amplified products. Kinetic traces of FAM fluorescence generation from three replicates for each condition are shown.

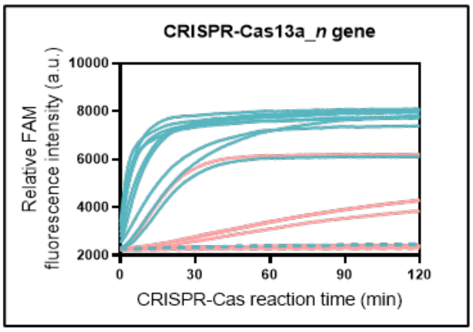

	Positive/Tested	
	NTC	Positive
Total reaction	0/3	9/9
Bulk phase	0/3	4/9

Supplementary Fig. 3. **More replicates of the Detection of amplified RPA product from the total RPA reaction vs the bulk phase solution.**

Following the preparation of the RPA reaction, aliquots were taken to represent the total reaction for analysis. The total RPA reaction was centrifuged to separate the bulk and droplet phases, and the resulting solutions were subjected to RPA amplification using 10,000 copies of the pUC57-2019-nCoV-N plasmid as DNA template. Negative controls (NTC) used RNAase-free water in lieu of DNA input. The LwaCas13a-based detection of the *n* gene of SARS-CoV-2 was performed on amplified RPA products from the total RPA reaction and the bulk phase. Kinetic traces of FAM fluorescence generation from nine replicates for the DNA template-containing reactions, and three replicates from the negative control reactions, are shown.

a

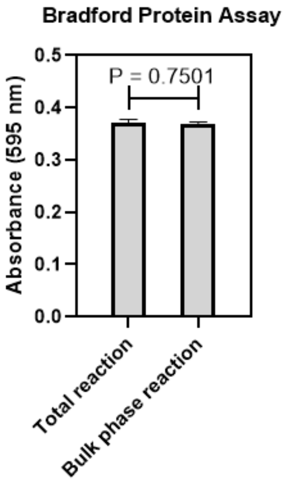

b

	**Total reaction (ng/µL)**	**Bulk phase (ng/µL)**	**Droplet phase (ng/µL)**
Negative condition	2379 ± 14	2418 ± 23	2470 ± 40
Positive condition	2546 ± 55	2468 ± 30	2673 ± 16

Supplementary Fig. 4**. a, Determination of protein concentrations in the total and bulk phase of RPA reactions**. Protein concentrations were measured using the Bradford assay. Data presented are means of absorbance ± s.d. from three replicates. No statistically significant difference was observed between the total reaction and the bulk phase reaction ($P = 0.7501$), as determined by Student's t-test at the 5% significance level. **b, Determination of DNA concentration in the total, bulk, and droplet phases after performing RPA reactions**. After completing the RPA reaction, aliquots were taken to represent the total reaction. The remaining amplified RPA products were centrifuged to separate the bulk and droplet phases. Subsequently, the amplified RPA products from the total reaction, bulk phase, and droplet phase were purified. DNA concentrations of the purified RPA products were measured using a NanoDrop spectrophotometer. Results are presented as three replicates for each group and are expressed as mean ± s.d. in the table. The high DNA concentrations present in the negative reactions are non-amplicon DNA species present in the reaction, particularly primers.

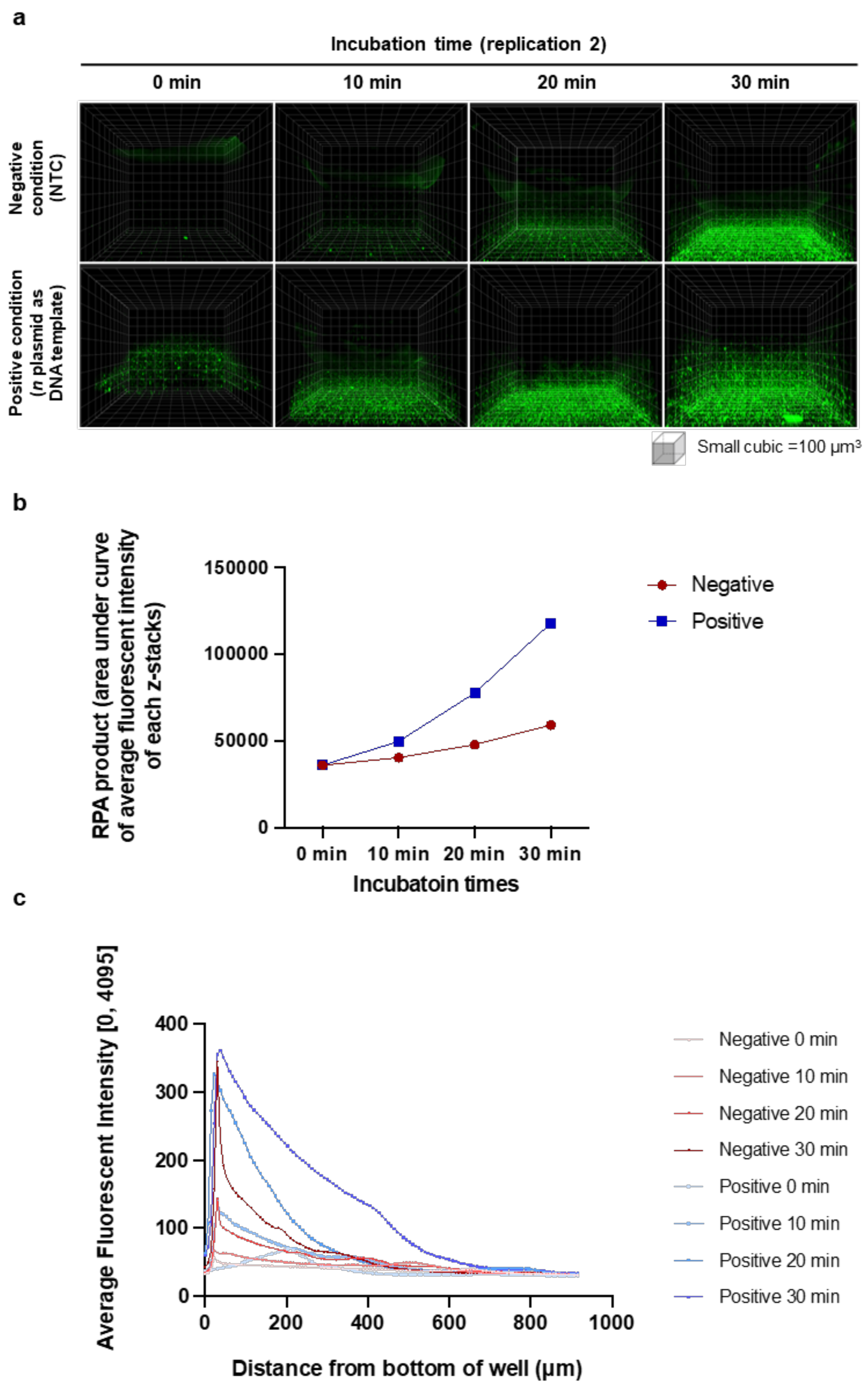

Supplementary Fig. 5**. An additional replicate of representative 3D confocal images of PicoGreen-stained RPA droplets**. **a,** Images from reaction time points of 0, 10, 20, and 30 min are shown for the *n* plasmid condition, and a no-template negative control condition. Voxel volume, 100 μm³**.** **b**, Quantification of PicoGreen staining of RPA droplets over time. Combined areas under the curve (AUC) of mean PicoGreen intensities from each Z-stack were plotted against RPA reaction times, a second replicate **c,** Mean PicoGreen fluorescence intensity as a function of Z-position (distance from the bottom of the well, μm), a second replicate.

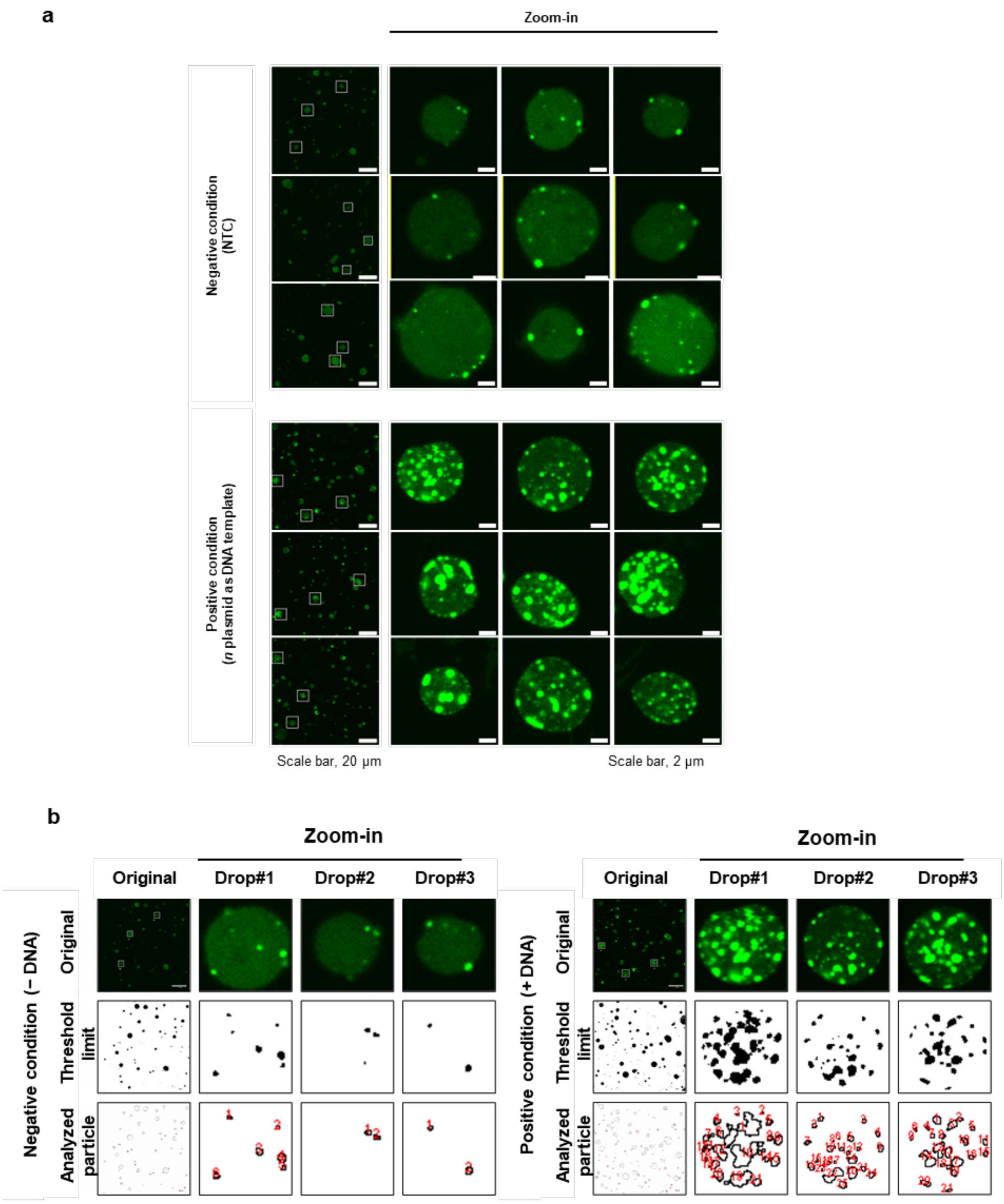

Supplementary Fig. 6**. Additional fields of view of high-magnification imaging with a 100X oil immersion objective of PicoGreen-stained RPA droplets.** Following completion of the RPA reaction, PicoGreen-based double-stranded DNA (dsDNA) staining was performed. **a**, Images for the template (*n* plasmid)-containing reaction and the no-template negative control are shown. **b**, PicoGreen-stained puncta were analyzed using the particle analysis feature in ImageJ software, with a lower size limit of 0.2 µm in diameter. To determine the area and apparent diameter of fluorescent droplets, the images were converted to an 8-bit format and subjected to thresholding using Fiji's built-in image thresholder. The Fiji particle analyzer algorithm, configured with a minimum detected size set to 0.2 µm² and circularity ranging from 0.2 to 1, was employed to identify circular objects and calculate their respective areas. These detected areas were then converted to apparent diameter values.

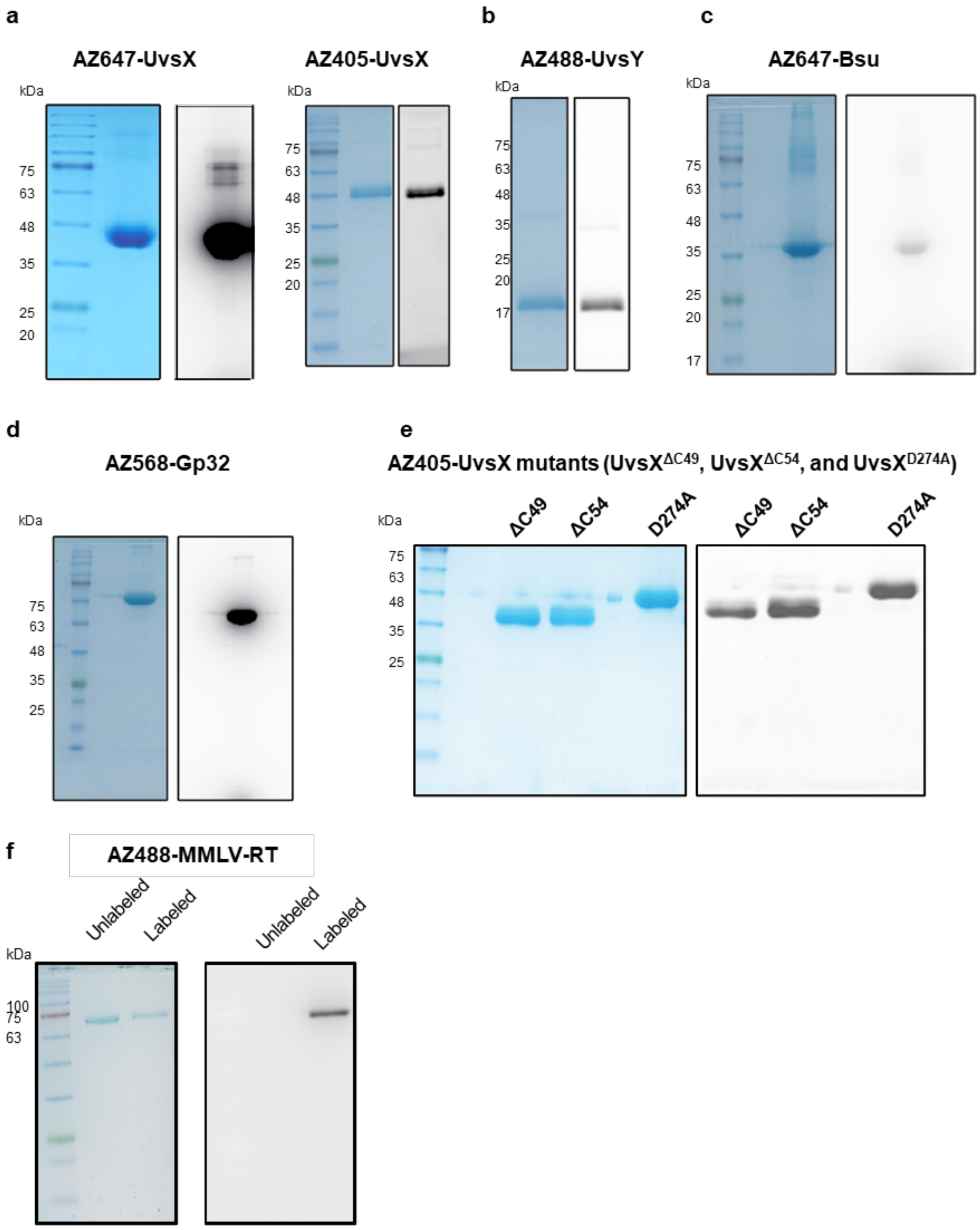

Supplementary Fig. 7. **SDS-PAGE of fluorescently labeled purified protein components of RPA and reverse transcriptase**

a-f, Fluorescently labeled RPA proteins and MMLV-RT were run on SDS-PAGE and their in-gel fluorescence visualized under the Amersham ImageQuant™ 800 Flour imager prior to Coomassie Blue staining. Calculated molecular weights for unlabeled proteins are as follows: UvsX 44.8 kDa; UvsY 17.5 kDa; Gp32 34 kDa; Bsu Pol 67.8 kDa; and MMLV-RT 74.6 kDa.

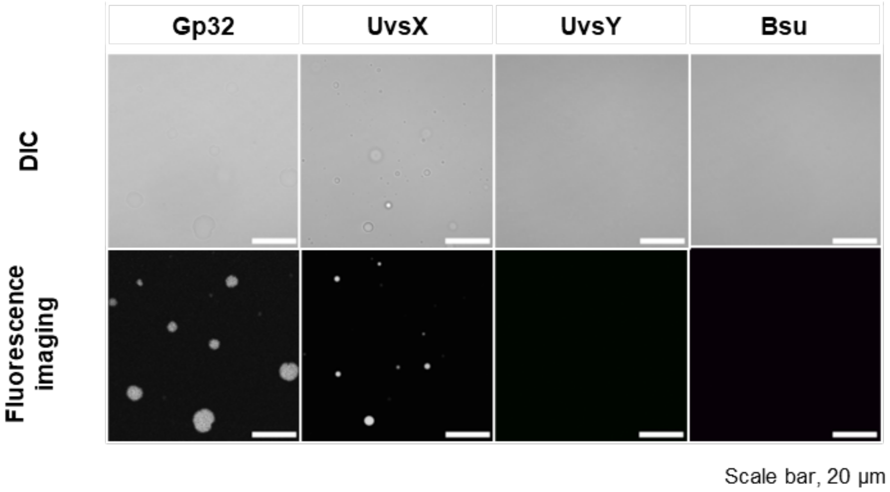

Supplementary Fig. 8. **Additional fields of view of phase separation of individual RPA proteins** (Fig. 3a)

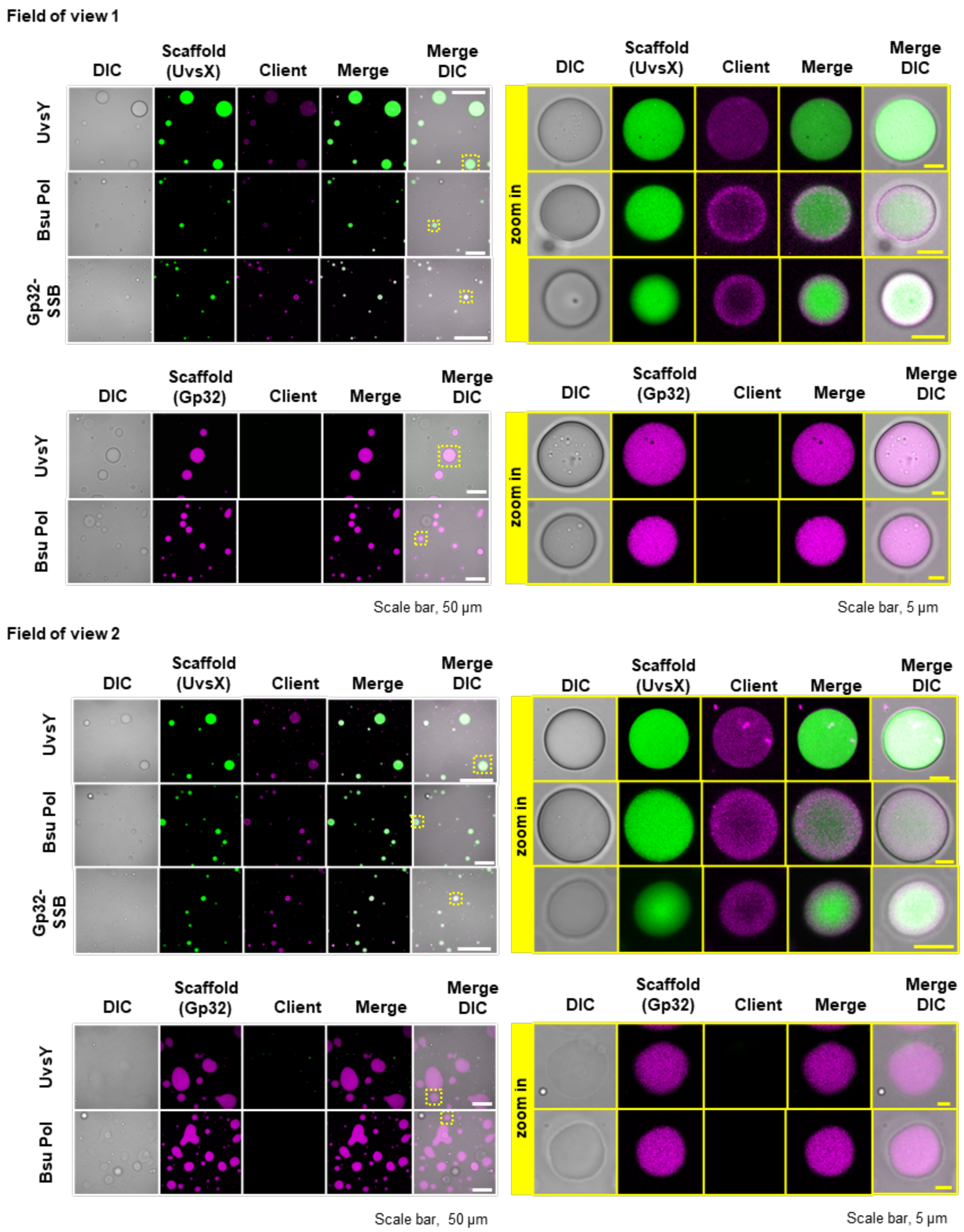

Supplementary Fig. 9. **Additional fields of view of condensates of dual RPA proteins** (Fig. 4)

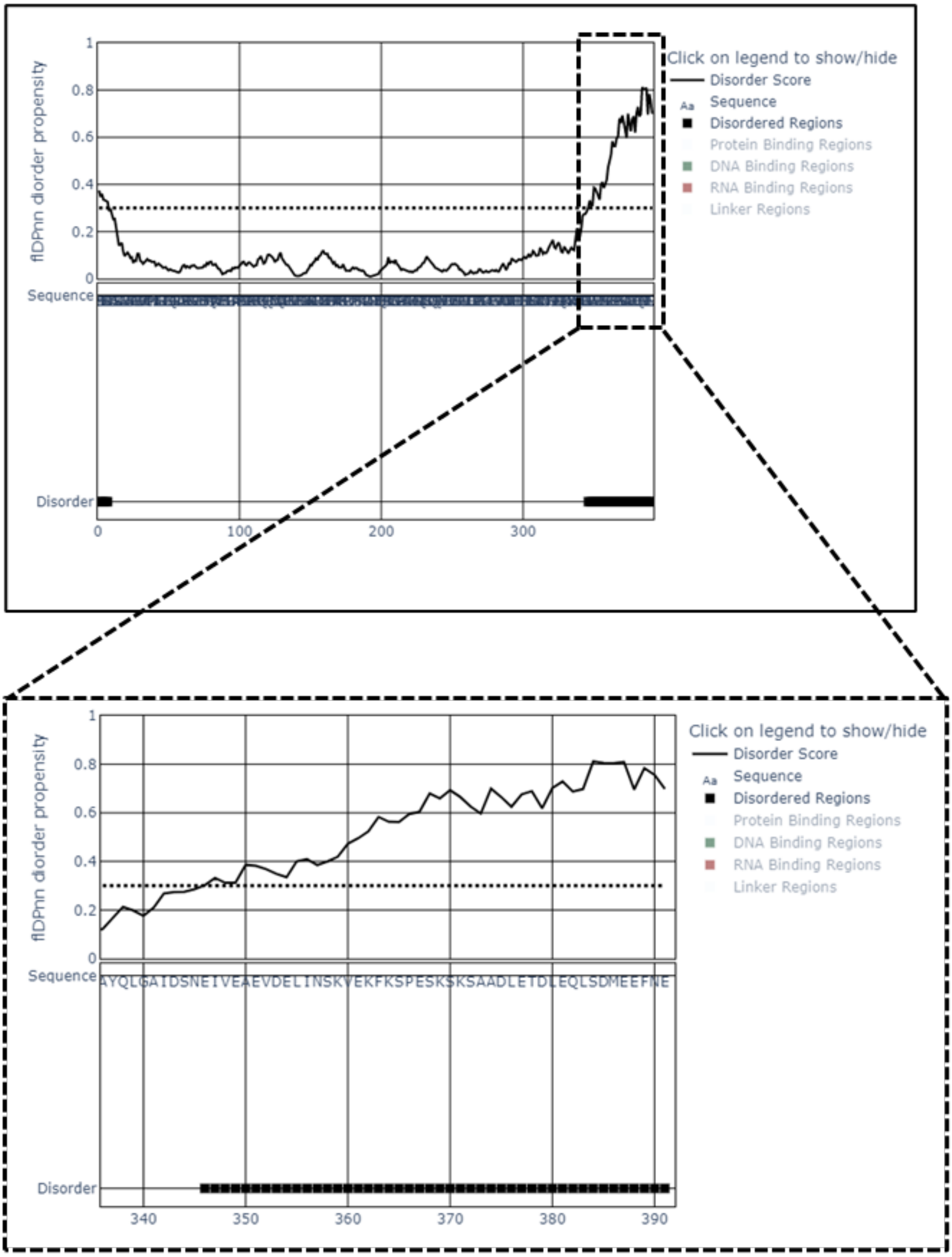

Supplementary Fig. 10. **Disordered region prediction of UvsX.** The amino acid sequence of UvsX was submitted to fIDPnn server[1], resulting in a disordered region prediction of UvsX, depicted as a black bar.

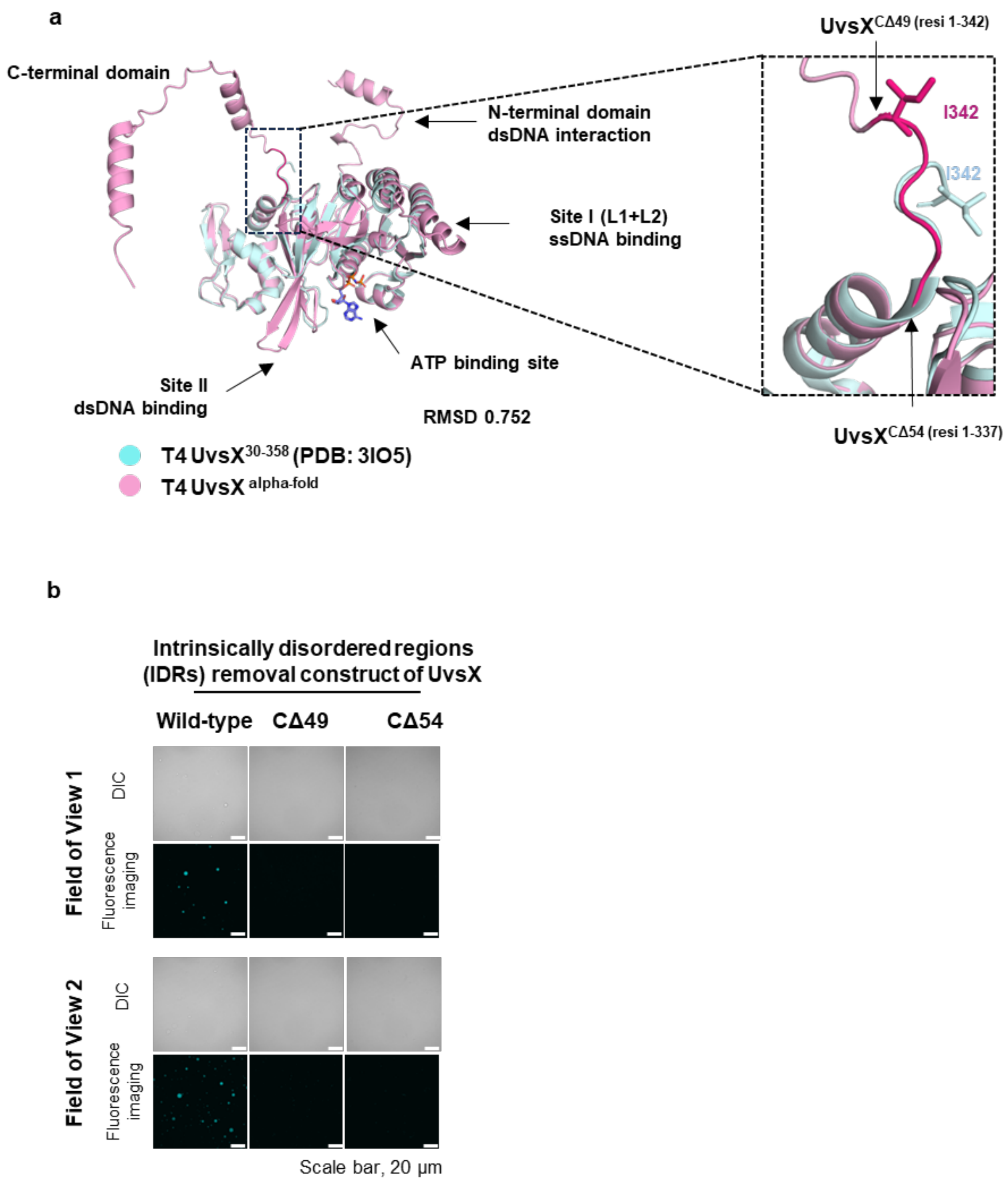

Supplementary Fig. 11. **a, comparison of the crystal structure and AlphaFold-predicted structure of T4 UvsX**. The experimental (cyan; PDB 3IO5) and AlphaFold-predicted (pink) structures of monomeric UvsX. The predicted structure contained the N- and C-termini of UvsX missing from the crystal structure. Intrinsically disordered regions within the C-terminus and truncation points for $UvsX^{C\Delta49}$ and $UvsX^{C\Delta54}$ were highlighted. **b, additional fields of view of comparison of UvsX variants**.

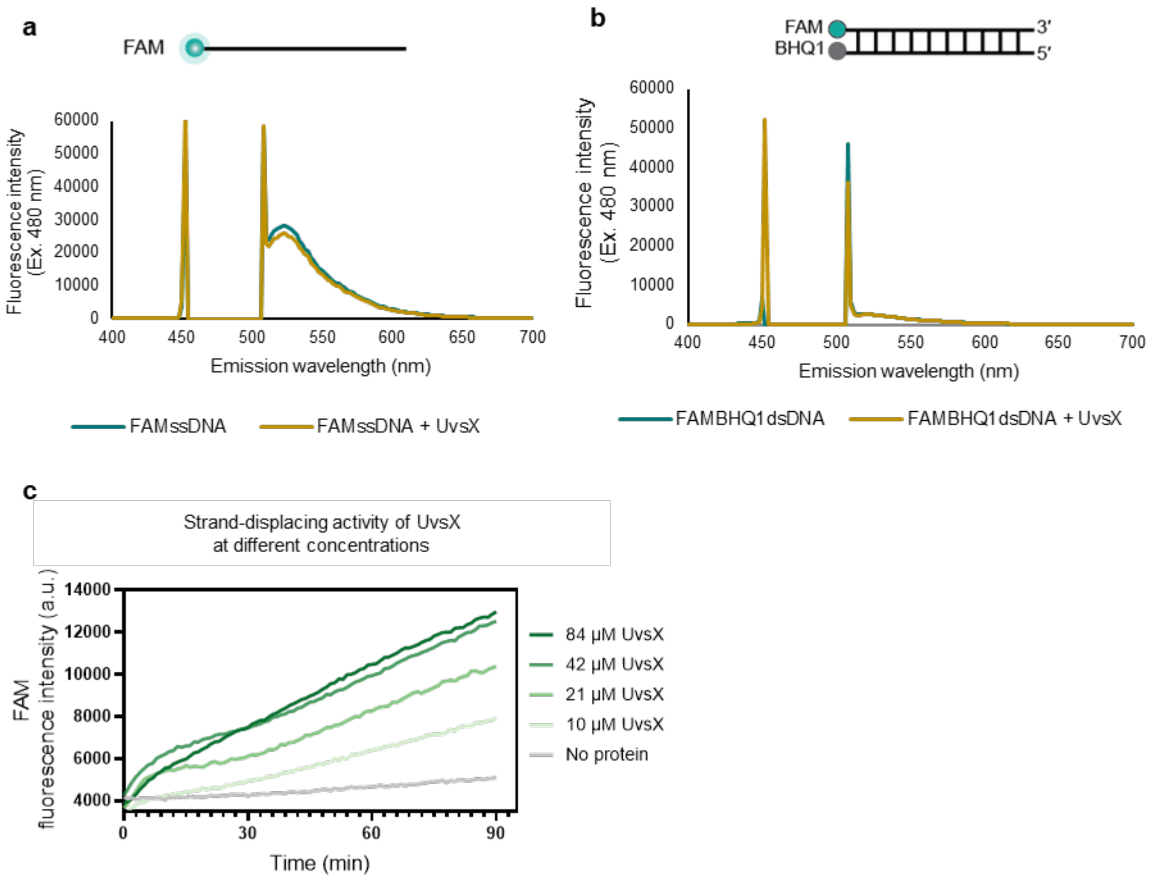

Supplementary Fig. 12. **Validation of FRET-based real-time measurements in the DNA strand displacement assay. a-b,** UvsX has no discernible effect on the fluorescence emission of FAM (a) or the quencher (b). Green spectra, FAM-labeled ssDNA (a) or FAM-BHQ1-labeled dsDNA (b) without UvsX. Yellow spectra, FAM-labeled ssDNA (a) or FAM-BHQ1-labeled dsDNA (b) with UvsX. **c,** Fluorescence emission of the released FAM-labeled ssDNA strand upon increasing concentrations of UvsX.

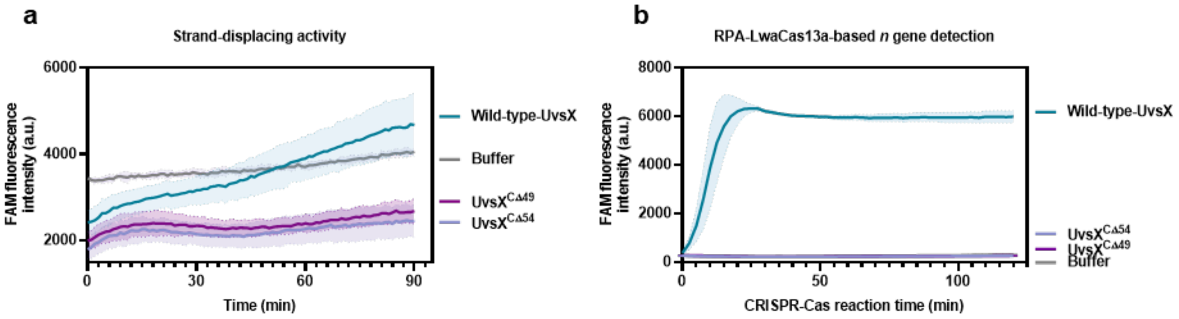

Supplementary Fig. 13. **UvsX activity assessment via strand displacement activity and CRISPR-based detection of RPA amplification reaction. a,** Time-coursed measurements of FAM fluorescence generation with UvsX variants. **b,** RPA amplification reaction using different UvsX variants, followed by LwaCas13a-based detection of the *n* gene. The template employed was 10,000 copies of the pUC57-2019-nCoV-N plasmid. Data presented are means ± s.d. from three replicates.

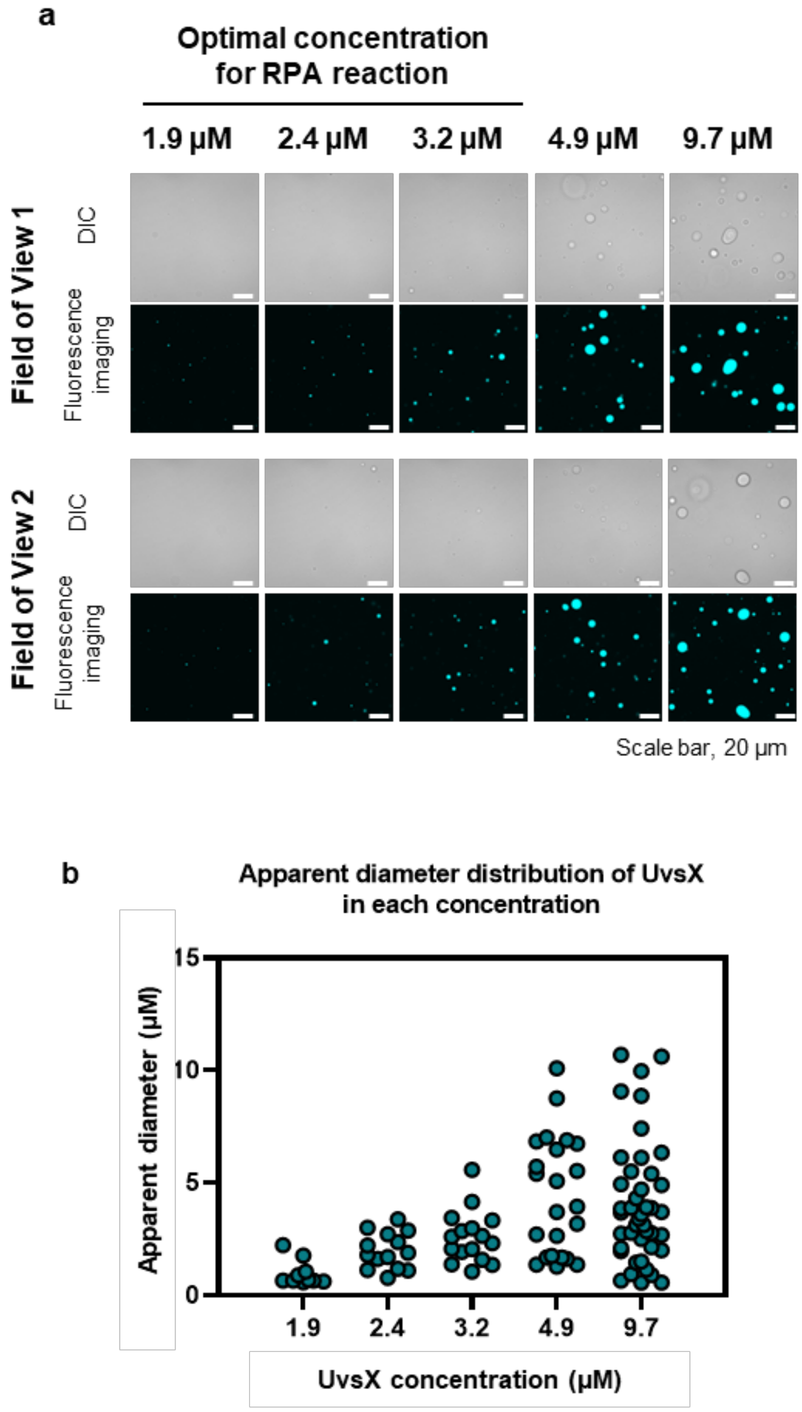

Supplementary Fig. 14. **a**, Phase separation of UvsX at increasing protein concentrations. Representative confocal images of A647-labeled UvsX at different concentrations are shown. Scale bar, 20 μm. **b,** Apparent diameters of UvsX droplets at varying UvsX concentrations in **a**. Image processing details are provided in the Methods section.

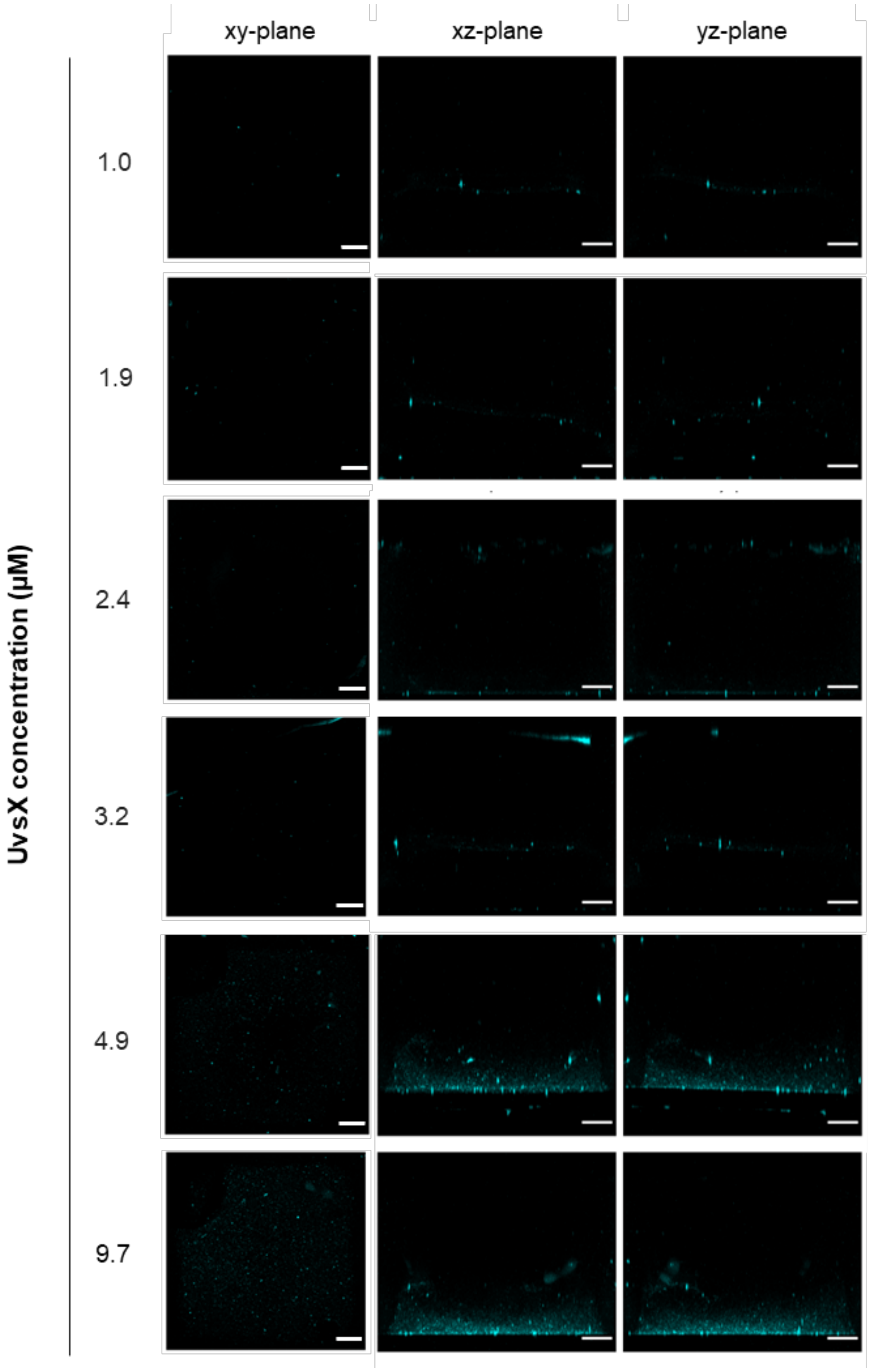

Supplementary Fig. 15. Representative maximum intensity projections of RPA droplets containing AZ405-labeled UvsX at different concentrations at 30 minutes after the reaction started, which were used to calculate droplet count and size distributions in Fig. 6b-c.

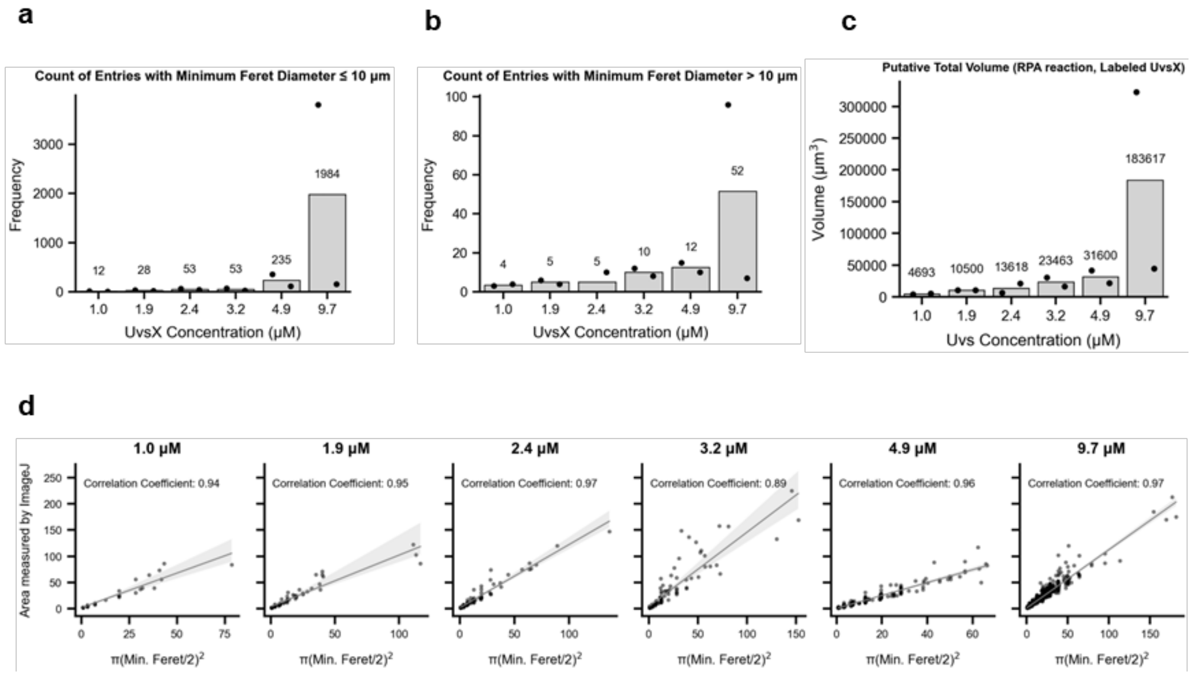

Supplementary Fig. 16. **RPA droplet count, size distribution, and volume estimation. a**, Count of droplets smaller or equal to 10 μm in the UvsX-AZ405 labeled RPA reactions at various UvsX concentrations. **b**, Count of droplets larger 10 μm in the UvsX-AZ405 labeled RPA reactions at various UvsX concentrations. **c**, Putative total volume of UvsX-AZ405 labeled RPA droplets calculated based on the minimum Feret diameter. **d**, Correlation coefficient between the droplet measurements obtained by ImageJ and the circular area approximation based on the minimum Feret diameter, demonstrating that the volume approximation using the minimum Feret diameter is a reasonable estimation.

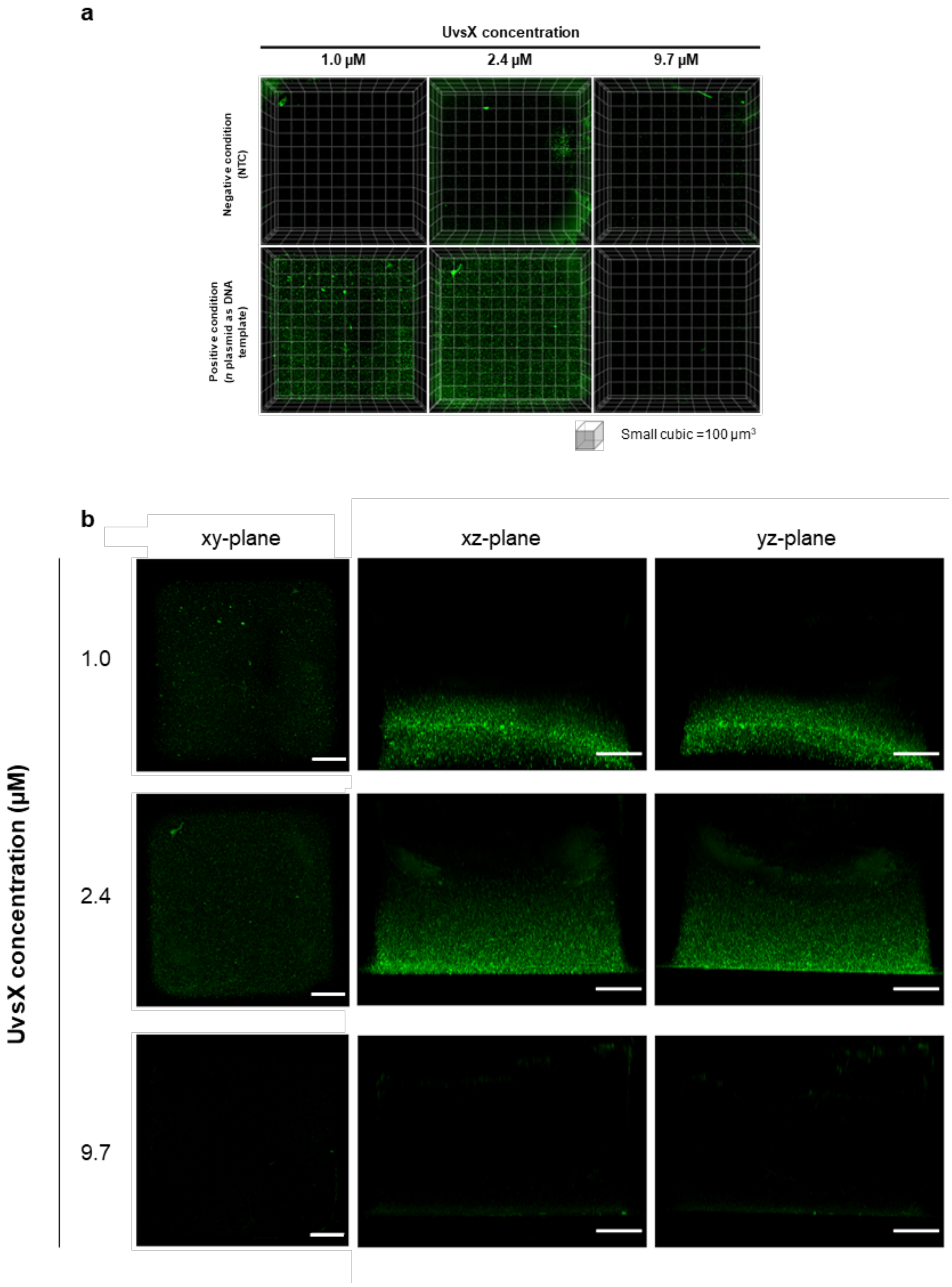

Supplementary Fig. 17. **PicoGreen staining of RPA droplets at various UvsX concentrations with negative controls**. **a**, RPA reactions were set up with UvsX at 1.0 µM, 2.4 µM, and 9.7 µM and incubated for 30 minutes. Positive conditions using the *n* gene plasmid as DNA template, while negative control used nuclease-free water. After the reaction, 10 µL of RPA products were stained with PicoGreen (final concentration 1X). For imaging, 1 µL of the stained mixture was loaded into a custom-fabricated 1 mm^3 well on an imaging dish, followed by a 10-minute settling period to allow all droplets to stabilize. Confocal imaging was performed using a 10x objective lens and 488 nm laser excitation. Z-series images (124 slices at 7.5 µm per slice, resolution 640x640 pixels) were captured from the bottom to the top of the well. The

3D structure of the droplets was reconstructed using Olympus FV31S-SW software. The top view of the 3D structure was cropped and zoomed to 125% to represent a 1 mm x 1 mm dimension. Small cubic grid lines, corresponding to 100 μm x 100 μm x 100 μm, were overlaid on the image as a reference for distance measurement.

In the positive condition (*n* plasmid as DNA template), an increase in PicoGreen staining was observed at 2.4 μM UvsX compared to 1.0 μM, indicating enhanced RPA reaction. However, at 9.7 μM UvsX, the PicoGreen signal was nearly absent, suggesting that the RPA reaction did not occur effectively. In the negative condition (NTC, non-template control), low PicoGreen signals were observed across all UvsX concentrations.

b, Representative maximum intensity projections of the PicoGreen-labeled RPA droplets at 15 minutes after the reaction started (single-color imaging)

a

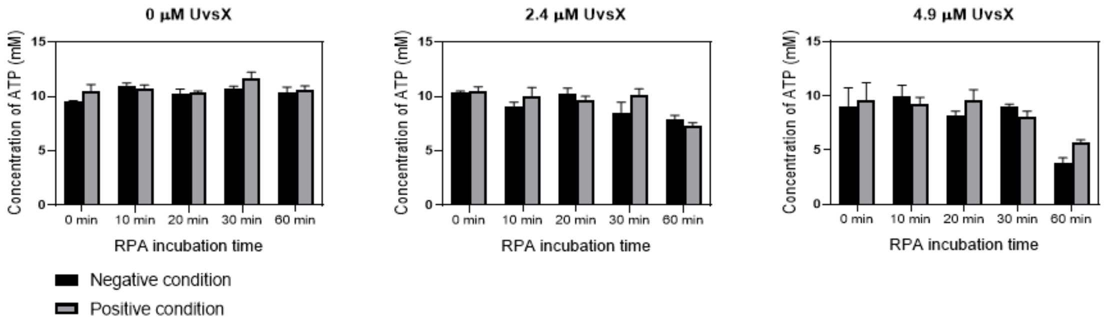

b

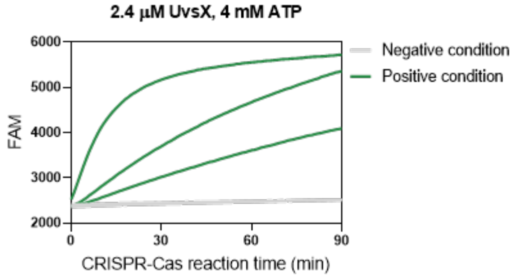

Interpretation of data

	Positive/Teste d
Negative condition (NTC)	0/3
Positive condition (pUC57-2019-nCoV-N plasmid)	3/3

Supplementary Fig. 18. **a, Determination of remaining ATP levels after the RPA reaction at different UvsX concentrations.** ATP levels were measured using a bioluminescent ATP assay based on the firefly luciferase enzymatic reaction. Measurements were taken at specified time intervals (0, 10, 20, 30, and 60 minutes post-reaction). The black bar represents the ATP level under negative conditions (non-template control with RNase-free water as input), while the grey bar represents the ATP level under positive amplification conditions (10,000 copies of pUC57-2019-nCoV-N plasmid used as input) (mean ± SD, n = 3). The remaining ATP concentration at each time was calculated from a standard curve. **b, LwaCas13a-based *n* gene of SAR-CoV-2 detection of amplified RPA product produced by the RPA reaction using 4 mM ATP.** Three replicates for the negative and positive conditions are shown. Data interpretation is provided.

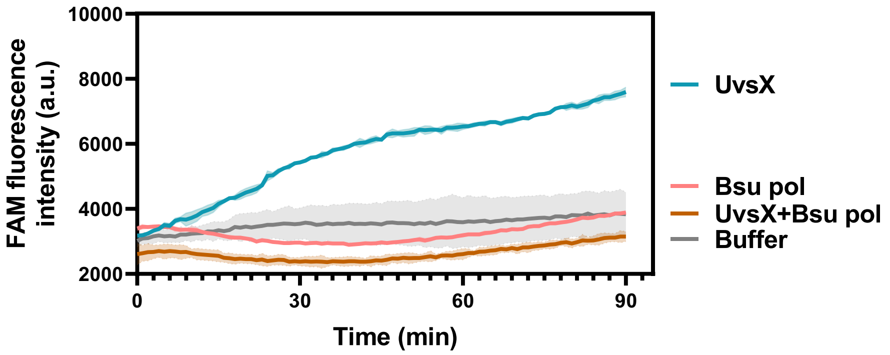

Supplementary Fig. 19. ***Bsu* DNA polymerase inhibits strand displacement by UvsX**. Raw data of FAM fluorescence generation by UvsX displacing FAM-ssDNA from Fig. 7a. Duplicates are showed as mean ± s.d.

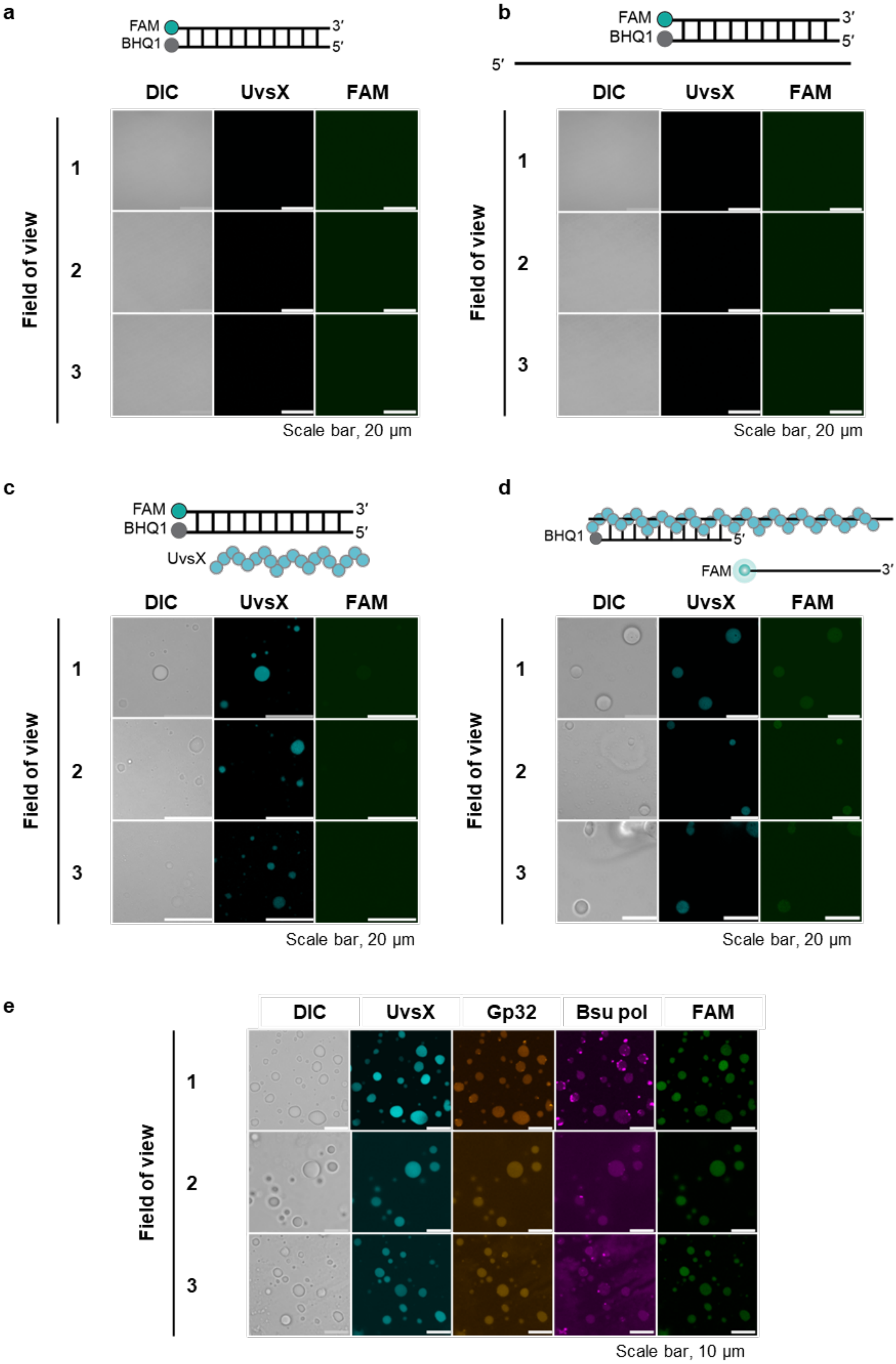

Supplementary Fig. 20. **Strand-displacing activity measurements in droplets**

a-d, Confocal imaging of strand-displacing activity facilitated by UvsX. The reactions were prepared with the following oligonucleotide and protein concentrations: 40 nM FAM-BHQ1 dsDNA; 40 nM unlabeled ssDNA; 3.3 μM UvsX with 0.2 μM AZ405-labeled UvsX for FAM-BHQ1 dsDNA with UvsX; and 18 μM UvsX with 0.2 μM AZ405-labeled UvsX for FAM-BHQ1 dsDNA and unlabeled ssDNA with UvsX. The mixtures were prepared in an RPA buffer (50 mM Tris-HCl pH 7.5, 100 mM KOAc, 14 mM MgOAc, 2 mM DTT, and 5% (w/v) PEG20000) supplemented with 2.5 mM ATP. Scale bar, 20 μm. **e,** Confocal imaging of strand-displacing activity facilitated by UvsX in the presence of other RPA proteins. The reactions were prepared with the following oligonucleotide and protein concentrations: 40 nM FAM-BHQ1 dsDNA; 40 nM unlabeled ssDNA; 3.3 μM UvsX with 0.2 μM AZ405-labeled UvsX; 3.3 μM UvsY; 26 μM Gp32 with 0.2 μM AZ568-labeled Gp32; and 1.8 μM Bsu Pol with 0.2 μM AZ647-labeled Bsu Pol. The mixture was prepared in an RPA buffer (50 mM Tris-HCl pH 7.5, 100 mM KOAc, 14 mM MgOAc, 2 mM DTT, and 5% (w/v) PEG20000) containing 2.5 mM ATP.

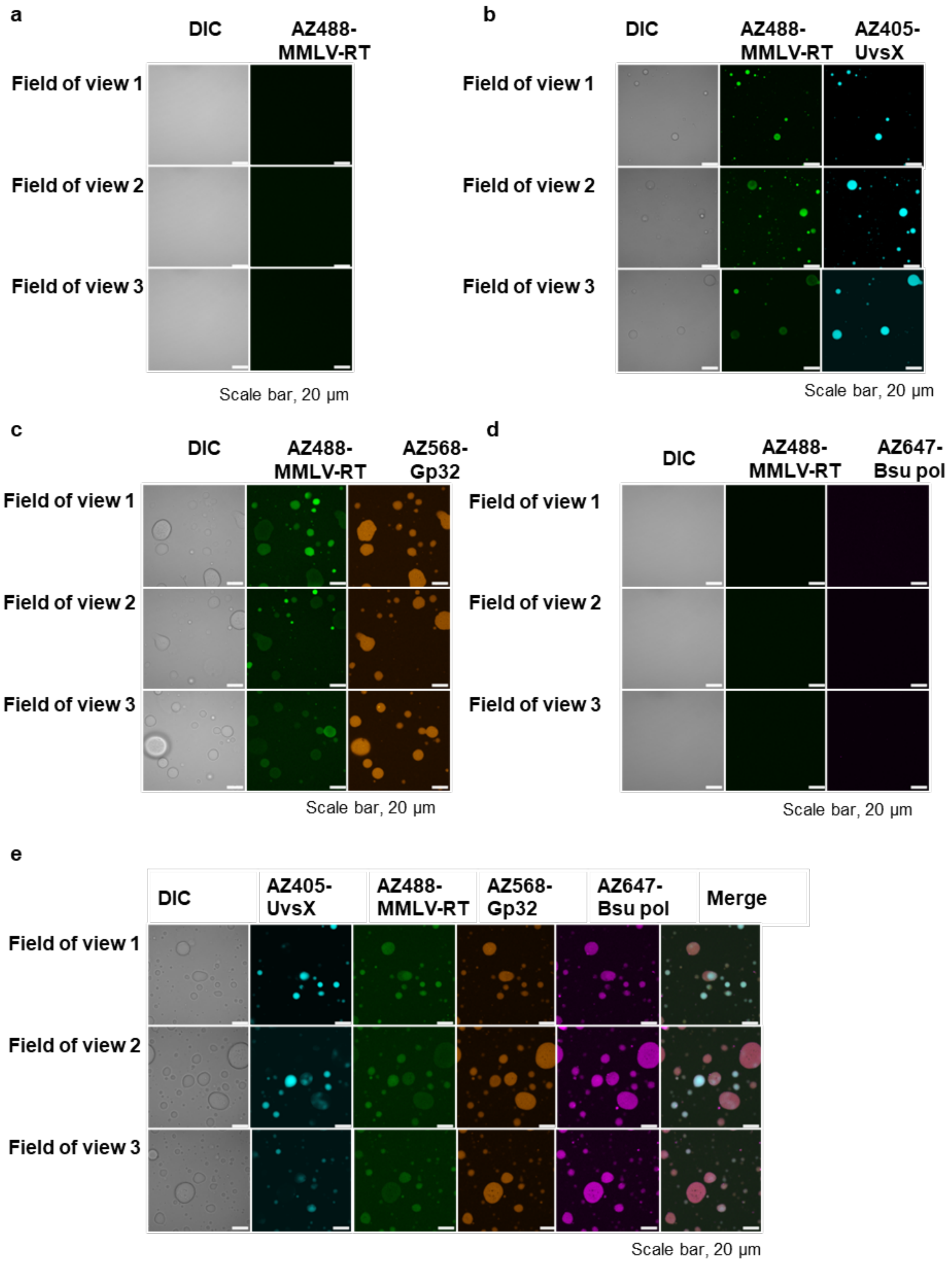

Supplementary Fig. 21. **Phase separation of MMLV-RT in combination with RPA proteins**

a, MMLV-RT by itself could not form condensates. **b,** UvsX could recruit MMLV-RT into condensates. **c,** Gp32 could recruit MMLV-RT into condensates. **d,** A mixture of RT and Bsu Pol did not form condensates. **e,** The combination of MMLV-RT with the four RPA proteins rarely resulted in the formation of multiphase condensates. The

concentrations of the mixed proteins are as follows: 3.3 μM UvsX with 0.2 μM AZ405-labeled UvsX; 3.3 μM UvsY; 26 μM Gp32 with 0.2 μM AZ568-labeled Gp32; and 1.8 μM Bsu Pol with 0.2 μM AZ647-labeled Bsu pol; and 0.1 μM RT and 0.3 μM AZ488-labeled RT. This mixture was prepared in an RPA buffer (50 mM Tris-HCl pH 7.5, 100 mM KCl, 14 mM MgOAc, 2 mM DTT, 5% (w/v) PEG20000). Scale bars, 20 μm.

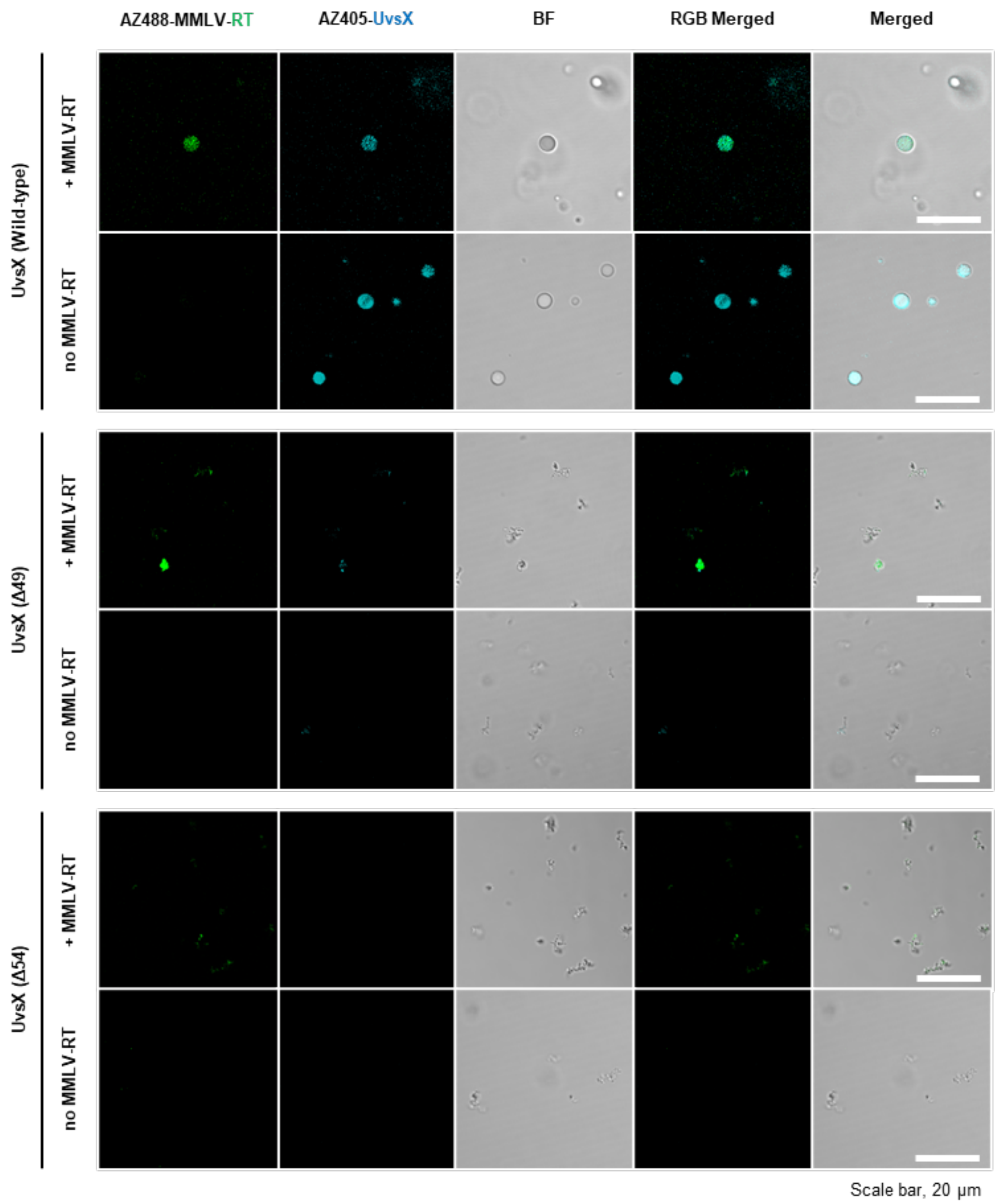

Supplementary Fig. 22. **Detection of droplet formation of UvsX truncation variants with and without MMLV-RT**. Confocal microscopy images were obtained to analyze the droplet formation in wild-type UvsX and its truncated mutants (UvsXΔ49 and UvsXΔ54). Fluorescence signals were captured using laser excitation at 405 nm and 488 nm. Droplet formation was observed in the wild-type UvsX but was absent in both truncated mutants, regardless of the presence or absence of MMLV-RT. These results suggest that the deleted regions in the UvsX mutants are critical for droplet formation. Images were captured using a 60× objective lens with oil immersion. Scale bar = 20 μm.

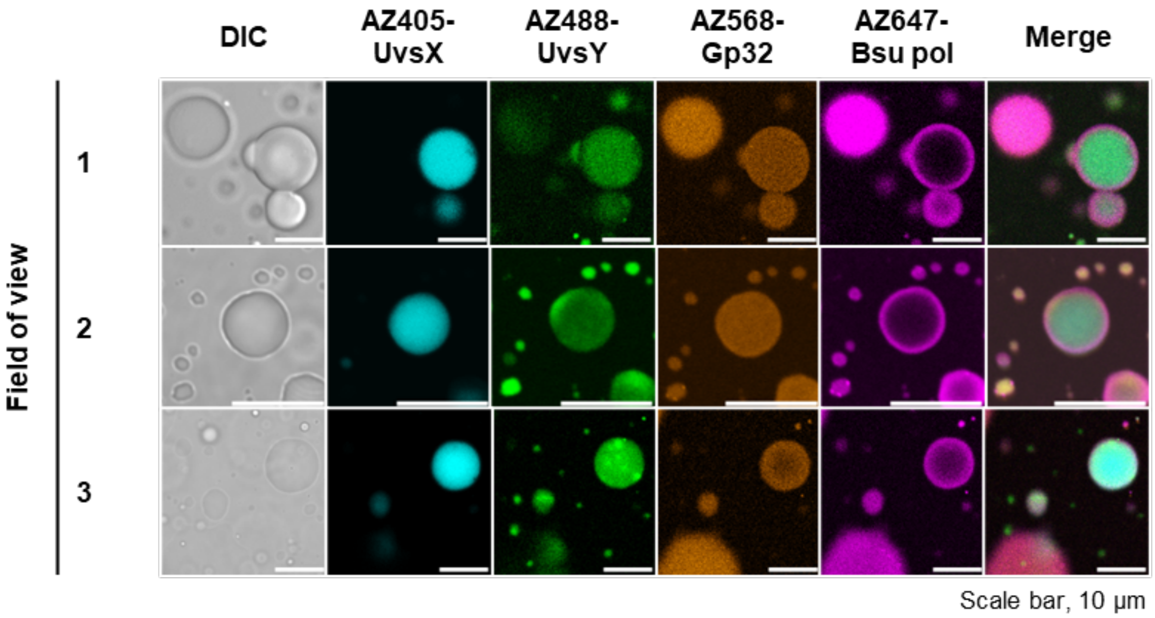

Supplementary Fig. 23. **Additional fields of view of RPA multiphase condensates** (Fig. 7c)

Sequence identity

	T4-UvsX	*E. coli* RecA	*D. Radiodurans* RecA
**T4-UvsX**	100		
***E. coli* RecA**	21.6	100	
***D. Radiodurans* RecA**	20.8	55.3	100

Sequence similarity

	T4-UvsX	*E. coli* RecA	*D. Radiodurans* RecA
**T4-UvsX**	100		
***E. coli* RecA**	40.3	100	
***D. Radiodurans* RecA**	35.9	70.0	100

Supplementary Fig. 24. **Sequence identity and similarity of UvsX and RecA.** Percent sequence identity and similarity of UvsX and RecA enzymes. Pairwise amino-acid alignments were performed using the EMBOSS-Needle method[2]. Species abbreviations and their accession number are as follow: *E. coli*, *Escherichia coli* RecA (WP_062896726.1); *D. radiodurans*, *Deinococcus radiodurans* RecA (AAF11887.1); T4-UvsX, Escherichia virus T4 UvsX (NP_049656.2).

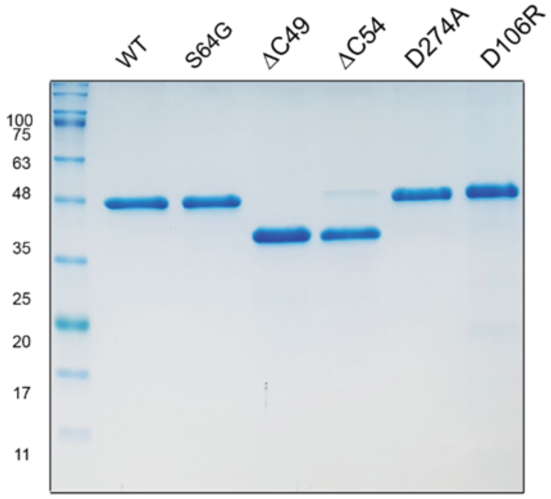

Supplementary Fig. 25. **Purity of UvsX mutants used in this study.** UvsX variants were recombinantly expressed in *E. coli* and initially purified using Ni-NTA column. Subsequently, each variant was subjected to the different steps of purification (detailed in the method section). Purified proteins were analyzed by SDS-PAGE followed by Coomassie Blue staining.

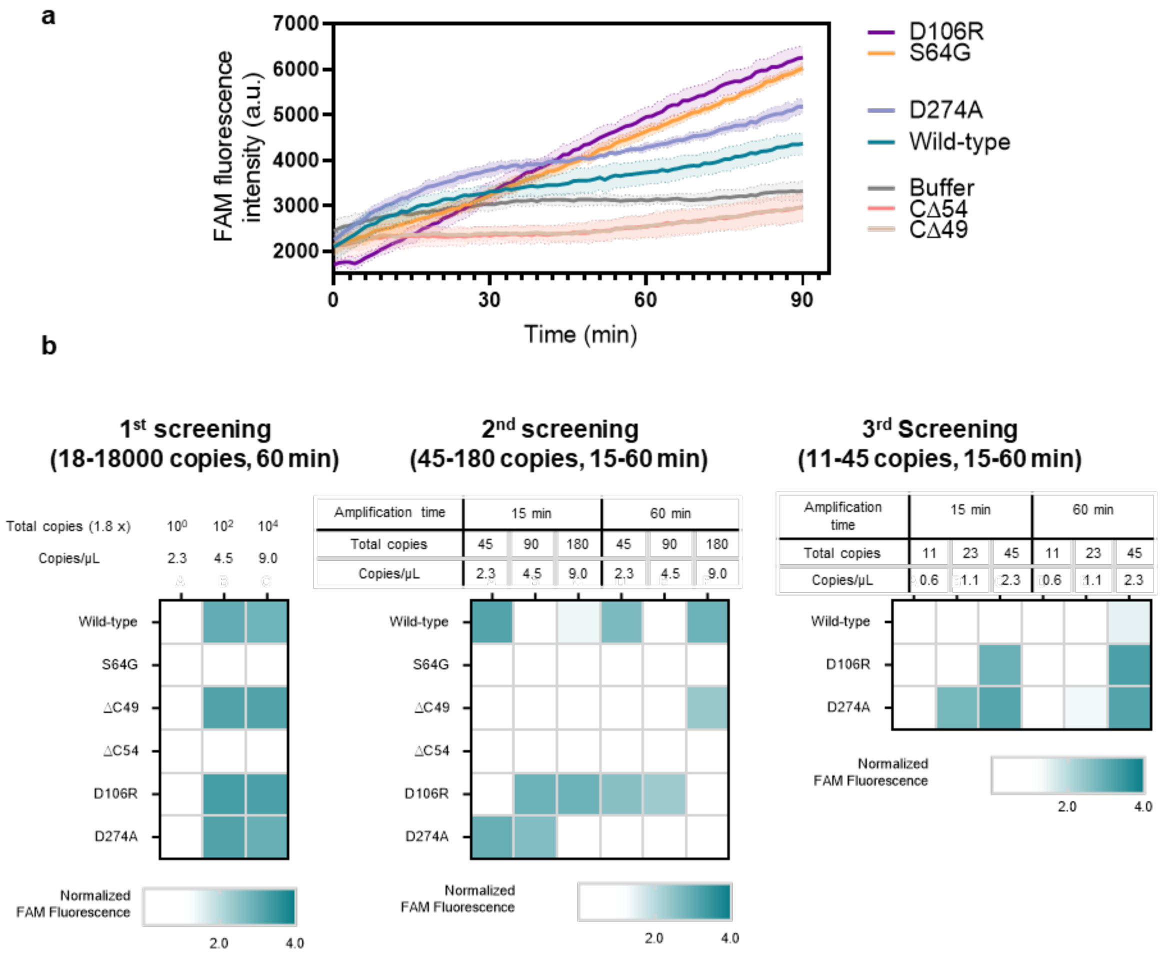

Supplementary Fig. 26. **Strand-displacing activity and RPA amplification of UvsX mutants**

a, Raw Kinetics of FAM fluorescence generation from UvsX variants displacing FAM-ssDNA from Fig. 8b. Triplicates are presented as mean ± s.d. **b,** Assessment of RPA activity by UvsX mutants. UvsX variants were initially screened with concentrated DNA input, then further challenged with more diluted DNA input amount in the second and third screening rounds. FAM fluorescence at 60 minutes were normalized against intensities obtained from the no template control. For these mutant screening experiments, we performed each experiment once and used the overall trend to pick the key mutant (D274A) for further characterizations.

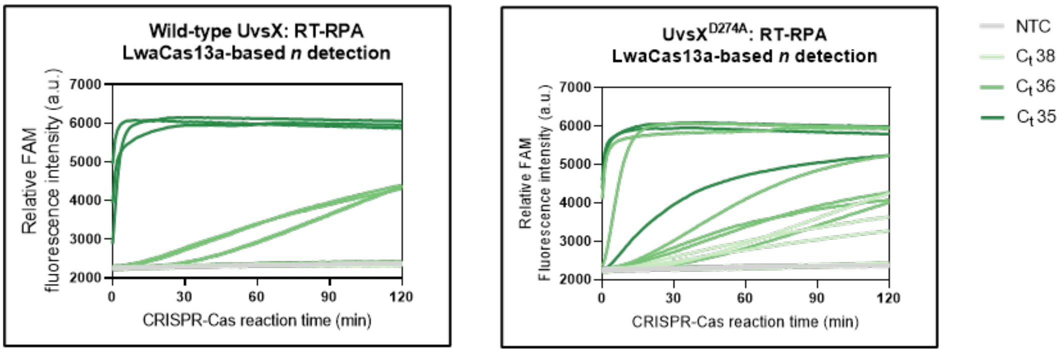

Supplementary Fig. 27. **RT-RPA amplification with wild-type UvsX and Uvsx^{D274A}.** Kinetic traces of FAM fluorescence generation over 120 min from Fig. 8c. Three replicates are showed as mean ± s.d. Three replicates of the amplification and detection reactions were performed for the negative control (NTC) and C_t 35, while ten replicates were performed for C_t 36 and C_t 38.

a

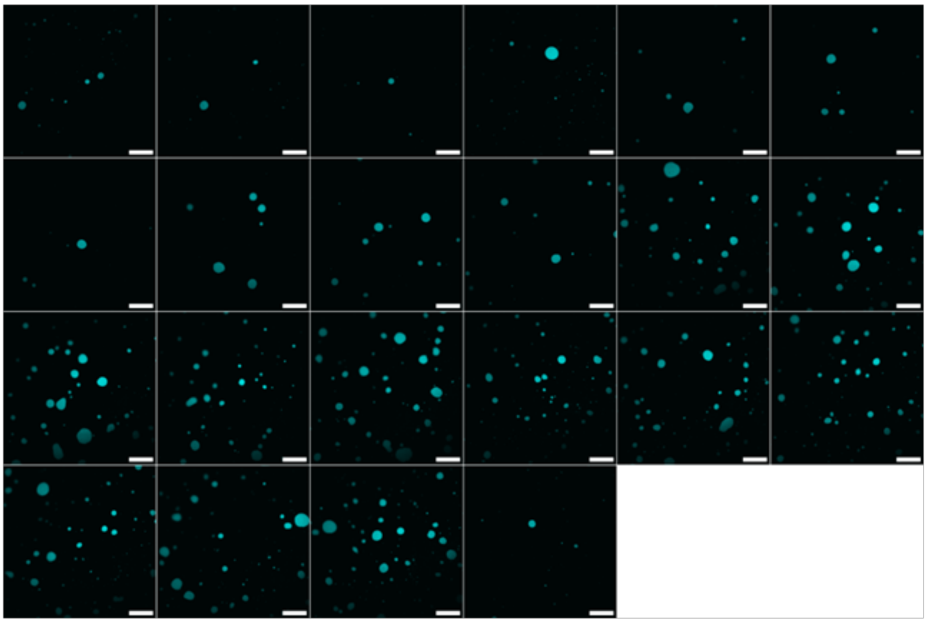

Scale bar, 20 µm

b

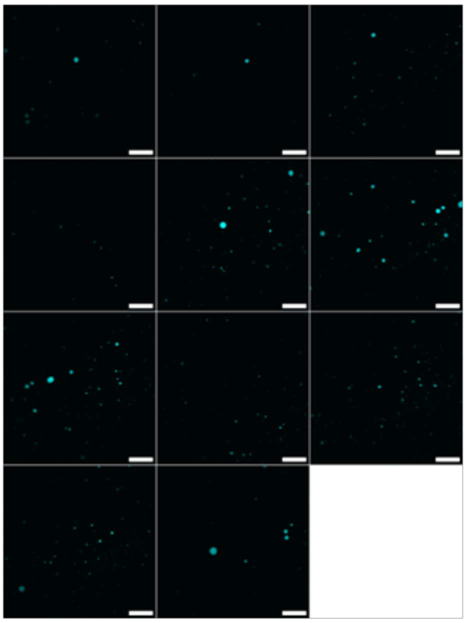

Scale bar, 20 µm

c

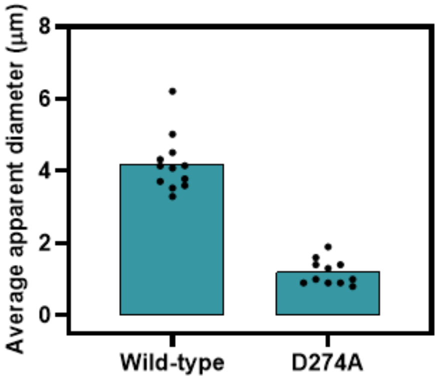

Supplementary Fig. 28. **Additional fields of view for the comparison of droplet sizes of wild-type UvsX vs UvsX^{D274A}** (Fig. 8d). Confocal fluorescence imaging of wild-type UvsX (**a**) and UvsX^{D274A} (**b**). Representative images were obtained in phase-separation experiments in samples containing 3.8 µM UvsX or UvsX^{D274A} and 0.3 µM AZ405-labeled UvsX or AZ405-labeled UvsX^{D274A}. **c**, The determined apparent diameter of droplets from multiple fields of view are shown.

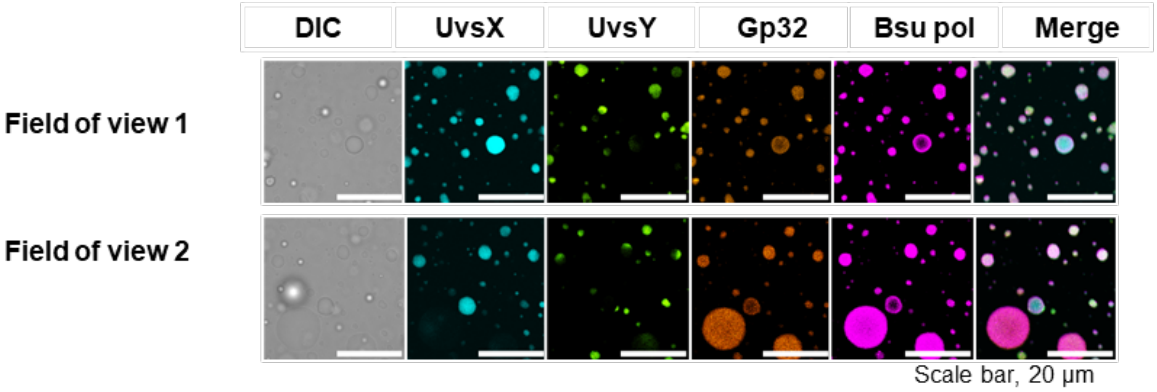

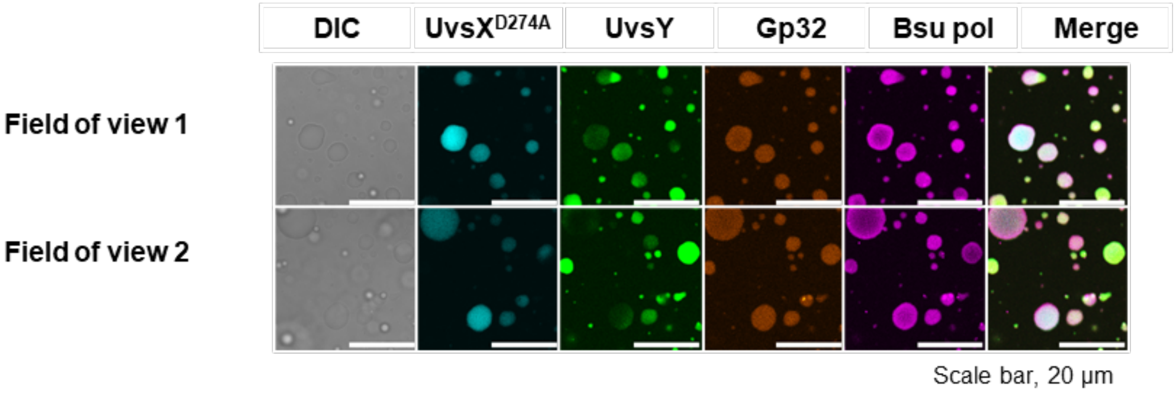

Supplementary Fig. 29. **Additional fields of view of RPA condensates with wild-type UvsX vs UvsX^{D274A}** (Fig. 8e)

Materials and methods

Tables

Table 1. Site-directed mutagenesis of T4 UvsX recombinase in this study

Bacteria RecA	T4 UvsX
E. coli RecA^{D112R}	UvsX^{D106R}
D. radiodurans RecA^{S82G}	UvsX^{S64G}
E. coli RecA$^{\Delta C25}$	UvsX$^{C\Delta 54}$
E. coli RecA$^{\Delta C17}$	UvsX$^{C\Delta 49}$
E. coli RecA^{D276A}	UvsX^{D274A}

Table 2. Primers for site-directed mutagenesis to create UvsX mutants

Name	Sequence 5'-3'	Source	Ref
UvsX-CΔ49-f	GCA TAT CAG CTG GGT GCC ATC CAC CAT CAC CAT CAC CAC TAA	IDT	This study
UvsX-CΔ49-r	TTA GTG GTG ATG GTG ATG GTG GAT GGC ACC CAG CTG ATA TGC	IDT	This study
UvsX-CΔ54-f	GAT GCC ATT AAA CGT GCA TAT CAC CAT CAC CAT CAC CAC TAA	IDT	This study
UvsX-CΔ54-r	TTA GTG GTG ATG GTG ATG GTG ATA TGC ACG TTT AAT GGC ATC	IDT	This study
UvsX-S64G-f	ATT CTG GCT GGT CCG AGC AAA **GGC** TTT AAA AGC AAT TTT GGT CTG	IDT	This study
UvsX-S64G-r	CAG ACC AAA ATT GCT TTT AAA **GCT** TTT GCT CGG ACC AGC CAG AAT	IDT	This study
UvsX-D106R-f	TAT CTG CGT AGC ATG GGT GTT **CGT** CCG GAA CGT GTT ATT CAT ACA	IDT	This study
UvsX-D106R-r	TGT ATG AAT AAC ACG TTC CGG **ACG** AAC ACC CAT GCT ACG CAG ATA	IDT	This study
UvsX-D274A-f	GAT CCG TAT AGT GGT CTG CTG **GCG** ATG GCA CTG GAA CTG GGT TTT	IDT	This study
UvsX-D274A-r	AAA ACC CAG TTC CAG TGC CAT **CGC** CAG CAG ACC ACT ATA CGG ATC	IDT	This study

Table 3. Oligonucleotides used in (RT)-RPA amplification followed by LwaCas13a-based detection of the SARS-CoV-2 *n* gene.

Name	Sequence 5'-3'	Source	Ref
**RT-RPA primer**			
SI-n-RPA-Forward_v4	gaaattaatacgactcactatagggGAACTTCTCCTGCTAGAATGGCTG	IDT	[3]
SI_n-RPA-Reverse_v1	CAGACATTTTGCTCTCAAGCTGGTTCAATC	IDT	[4]
**crRNA and Cas reporter**			

FAM PolyU reporter	/FAM/rUrUrUrUrC/IABkFQ/	IDT	[4]
13a_SI_n-crRNA_v1	gauuuagacuaccccaaaaacgaaggggacuaaaacAAAGCAAGAGCAGCAUCACCGCCAUUGC	Synthego	[4]

Table 4. Oligonucleotides in strand displacing assay

Name	Sequence 5'-3'	Source	Ref
FAM_N-80-F	**[6-FAM]**GGAACTTCTCCTGCTAGAATGGCTGGCAATGGCGGTGATG	BIONICS	This study
N-80-R_BHQ1	CATCACCGCCATTGCCAGCCATTCTAGCAGGAGAAGTTCC-**[BHQ-1]**	BIONICS	This study
N-80-F	ggaacttctcctgctagaatggctggcaatggcggtgatgctgctcttgctttgctgctgcttgacagattgaaccagct	BIONICS	This study

Methods

Sources and cloning methods for (RT)-RPA expression plasmids and UvsX mutants

Expression plasmids for protein components of RPA were previously reported and are available on Addgene: pET28a-MH6-Bsu LF (Plasmid #163911); pET28a-3gp32-H6 (Plasmid #163912); pET28a-uvsX-H6 (Plasmid #163913); and pET28a-MH6-uvsY (Plasmid #163914). pET21b-MMLV-RT (mutH)-His6 was subcloned via amplification of the coding sequence of the MMLV-RT (mutH) gene (sourced from The FreeGenes Project – stanford.freegenes.org) into pET21b at the NdeI/XhoI sites. Expression plasmids encoding UvsX mutants were generated via site-directed mutagenesis on pET28a-uvsX-H6, using primers in Table 2.

Protein expression and purification

Expression and purification of UvsX were previously described[3]. An expression plasmid for wild-type UvsX or its mutants were transformed into *E. coli* BL21(DE3) cells. The cells were grown in LB medium at 37 °C until OD_{600} reached 0.7–0.8. Protein expression was induced using the 1 mM isopropyl-β-D-thiogalactopyranoside (IPTG). The cells were grown for 16 hours at 16 °C and harvested by centrifugation. The cell pellet was resuspended in lysis buffer (50 mM sodium phosphate pH 8.0, 500 mM sodium chloride, 10 mM imidazole). Phenylmethylsulfonyl fluoride (PMSF) was added into the resuspended solution at 1 mM final concentration, followed by sonication for 3 minutes of total burst time (3 seconds on for short burst and 9 seconds off for cooling). The cell lysate was clarified by centrifugation at 27,000×*g* for 30 minutes at 4 °C. Nickel-nitrilotriacetic acid (Ni-NTA) agarose beads (Qiagen) were washed with two column volumes of water followed by equilibration with the lysis buffer. The clarified cell lysate was incubated with the Ni-NTA agarose beads (Qiagen) at 4 °C for 40 minutes. Ten column volumes (CV) of the washing buffer (50 mM sodium phosphate pH 8.0, 500 mM sodium chloride, and 20 mM imidazole) was flowed into the column. Bound proteins were eluted with the elution buffer (50 mM sodium phosphate pH 8.0, 500 mM sodium chloride, 250 mM imidazole). The protein was further purified by Heparin Sepharose Fast Flow (GE Healthcare Life Sciences) with a linear gradient of 0.1–1 M NaCl in Buffer A (20 mM Tris-HCl pH 8, 5 mM β-mercaptoethanol (βME)). Proteins were concentrated and stored in 20 mM Tris-HCl

pH 8.0, 500 mM NaCl, and 40% (v/v) glycerol at −20 °C. All proteins were determined to be >98% pure based on SDS-polyacrylamide gels stained with Coomassie Blue.

UvsY, Gp32, and *Bsu* polymerase proteins were expressed and purified as previously described[3]. For MMLV-reverse transcriptase, we used the same expression conditions in *E. coli* BL21(DE3) as UvsX expression. To purify MMLV-RT, cell pellets were resuspended in lysis buffer (20mM Tris-HCl pH 8.0, 300 mM NaCl, 0.5% (v/v) Triton X-100, 10% (v/v) glycerol, and 25 mM imidazole). The resuspended cells underwent sonication with 30 cycles of 5 seconds on and 9 seconds off, followed by centrifugation at 15,000 rpm for 30 minutes. The resulting supernatants were loaded into a 5 mL Histrap FF column, washed with a buffer containing 20mM Tris-HCl pH 8.0, 300 mM NaCl, 0.5% (v/v) Triton X-100, 10% (v/v) glycerol, and 80 mM imidazole, and eluted with a solution comprising 20mM Tris-HCl pH 8.0, 300 mM NaCl, 0.5% (v/v) Triton X-100, 10% (v/v) glycerol, and 250 mM imidazole. Subsequently, a Hitrap SP HP cation exchange chromatography column was used. The solution was diluted to establish an initial salt concentration of 50 mM NaCl at pH 6.5, followed by elution in a NaCl gradient of 50-500 mM. The resulting fractions were concentrated using Amicon 30 centricons, with the final salt concentration adjusted to a storage buffer (50mM Tris-HCl pH 8.0, 100 mM NaCl, 1 mM EDTA, 5 mM DTT, 50% glycerol), and then stored at −20 °C until use. The purified enzymes demonstrated a minimum purity level of 95%, as confirmed by SDS-PAGE analysis.

UvsX activity assessment through RPA or RT-RPA, followed by CRISPR-Cas13a-based reaction

The singleplexed RPA of the SARS-CoV-2 *n* gene was set up with the following components (20 μL total reaction volume, consisting of 19 μL reagent mastermix and 1 μL of DNA input): 50 mM Tris (pH 7.5), 100 mM potassium acetate, 14 mM magnesium acetate, 2 mM DTT, 5% (w/v) PEG20000, 200 μM dNTPs, 12 mM ATP, 50 mM phosphocreatine, 100 μg/mL creatine kinase, 150 μg/mL (3.3 μM) the wild-type UvsX or UvsX mutant, 30 μg/mL (1.7 μM) UvsY, 900 μg/mL (26.5 μM) Gp32, 120 μg/mL (1.8 μM) *Bsu* LF, 40 mM triglycine (Sigma), and 700 nM *n* gene primers (the F4/R1 pair). One μL of pUC57-2019-nCoV-N plasmid (MolecularCloud cat no. #MC_0101085) was used as a template, and the RPA reactions were allowed to proceed at 42 °C for 60 min.

The singleplexed RT-RPA of the *n* gene was set up with the following components (20 μL total reaction volume, consisting of 14.5 μL reagent mastermix and 5.5 μL RNA input): 50 mM Tris (pH 7.5), 100 mM potassium acetate, 14 mM magnesium acetate, 2 mM DTT, 5% (w/v) PEG20000, 200 μM dNTPs, 12 mM ATP, 50 mM phosphocreatine, 100 μg/mL creatine kinase, 150 μg/mL (3.3 μM) UvsX, 30 μg/mL (1.7 μM) UvsY, 900 μg/mL (26.5 μM) Gp32, 120 μg/mL (1.8 μM) *Bsu* LF, 26.6 U/mL RNase H, 2.8 U/μL EpiScript Reverse Transcriptase, 40 mM triglycine (Sigma), and 700 nM N gene primers. 5.5 μL of serially diluted SARS-CoV-2 RNA was used as a template, and the RT-RPA reactions were allowed to proceed at 42 °C for 60 min.

After (RT)-RPA, 2 μL of the RPA products were mixed with 18 μL of the LwaCas13a-based detection reaction, which contained 40 mM Tris-HCl pH 7.4, 60 mM NaCl, 6 mM $MgCl_2$, 1 mM of each rNTPs, 1.5 U/μL NxGen T7 RNA polymerase, 6.3 μg/mL LwaCas13a, 1 ng/μL LwaCas13a crRNA for N gene, and 0.3 μM FAM-PolyU reporter. The generated FAM fluorescence was monitored at 37 °C over 90 min using a

fluorescence microplate reader (Varioskan, Thermo Scientific, or Infinite M Plex, Tecan). The sequences of primers and crRNAs are listed on Table 3.

FRET-based measurements of DNA strand displacement

The oligonucleotides used for the strand displacement assay are listed in Table 4. To prepare the fluorescently quenched DNA duplex substrate, a 5′ FAM-labeled oligo (FAM_N-80-F) was annealed with its 3′-BHQ1 quencher labeled complementary strand oligo (N-80-R_BHQ1) at an equimolar amount of oligos in annealing buffer (10 mM Tris-HCl pH 7.5, 50 mM NaCl, and 1 mM EDTA). The mixture was heated at 95 °C for 5 min and gradually cooled to room temperature.

The concentration of all components in FRET-based real-time measurements of DNA strand displacement were stated as their final concentration in 20 μL total reaction volume. The reactions were conducted in a strand displacing buffer (50 mM Tris-HCl, pH 7.5, 100 mM potassium acetate, 14 mM magnesium acetate, 2 mM DTT, 5% (w/v) PEG20000, and 2.5 mM ATP) and RPA condition (1.7 μM UvsY, 26.5 μM Gp32, 1.8 μM *Bsu* DNA polymerase, 12 mM ATP, 50 mM phosphocreatine, 0.1 μg/μL creatine kinase, 200 μM dNTPs, 100 mM potassium acetate, 5% (w/v) PEG20000, 50 mM Tris-HCl pH 7.5, 2 mM DTT, 14 mM magnesium acetate). The presynaptic filament was formed by incubating 40 nM unlabeled ssDNA with UvsX (at the indicated concentrations) in a strand-displacing buffer. The reaction was then transferred into a 384-well microplate, and the reaction was started by adding 40 nM dsDNA substrate (5′ FAM-labeled dsDNA- BHQ1-3′) to the presynaptic complex solution. The change in FAM fluorescence was monitored at 530 nm upon excitation at 480 nm in a fluorescence microplate reader (Varioskan, Thermo Scientific or Infinite M Plex, Tecan) at a constant temperature of 37 °C for 90 minutes.

Fluorescent labeling of UvsX, UvsY, Gp32, *Bsu* Pol and MMLV-RT

AZDye™ 405/488/568/647 NHS Ester was purchased from Click Chemistry Tools. The stock solutions of AZDye™ 405 (17.2 mM), AZDye™ 488 (19.8 mM), AZDye™ 568 (15.8 mM), and AZDye™ 647 (12.8 mM) NHS Ester in DMSO were prepared by resuspending the lyophilized fluorescent dyes with DMSO to a concentration of 12.5 mg/mL. A 5-fold molar excess of AZDye™ over protein was added to 1.5 mg/mL of protein in a reaction buffer (20 mM HEPES pH 7.0, 250 mM NaCl). The solution was incubated in the dark at room temperature for 90 minutes, and excess fluorescent dye was removed using MicroSpin G-25 desalting columns (Cytiva), then concentrated. Finally, the proteins were stored in 20 mM HEPES pH 7.0, 250 mM NaCl, and flash-frozen in liquid nitrogen. The fluorescent dye-protein conjugates were analyzed on SDS-PAGE, which revealed a single band under the Amersham ImageQuant™ 800 Flour imager in the appropriate channel specific to the fluorophore prior to Coomassie Blue staining. Upon staining, the band appeared to run at approximately the same molecular weight as the unlabeled proteins. The labeling stoichiometry of each labeled protein was calculated from the absorbance of the fluorescence dye as indicated in the manufacturer's instructions and absorbance at 280 nm.

Protein concentration measurements for the total vs bulk phase of the RPA reaction

The Bradford method with Bio-Rad Protein Assay Dye Reagent Concentrate (Bio-Rad) was used. 45 μL of the total or bulk phase of the RPA reaction was mixed with

45 µL of the dye reagent (diluted per manufacturer's instructions) in 96-well plate well formats. The plate was incubated at room temperature for 5 minutes before measurements of absorbance at 570 nm using an Infinite M Plex microplate reader.

Time-coursed ATP concentration measurements of the RPA reaction
The reagents for ATP concentration measurements were prepared as follows: 1.25 µM luciferase enzyme; 6 mM $MgCl_2$; and 50 µM luciferin. After the RPA reactions were set up, a portion was removed at specified time intervals (0, 10, 20, 30, and 60 min post-reaction) and diluted 200-fold. 2 µL of diluted RPA products was mixed with 80 µL of the ATP concentration measurement reagent for 2 min in a white 96-well plate, and incubated for additional 5 min to stabilize the luminescence signal. Luminescence signals were recorded using a microplate luminometer with an integration time of 0.25-1 second per well. ATP concentrations in each sample were calculated from the standard curve generated from serially diluted ATP standards subjected to the same luciferase-based reagent.

Custom imaging chambers for volumetric imaging of RPA reactions
Customized imaging chambers were made using a 3D-printed mold and soft lithography with Polydimethylsiloxane (PDMS) as elastomeric material. The mold was designed using CAD software Autodesk Fusion 360. The design consisted of a central circular chamber with 20 mm diameter and 1 mm depth surrounded by 2 mm-thick wall and 3 mm-thick base. Inside the central chamber, four pillars were placed in each of the quadrant, with each pillar having a square base of 1 x 1 mm in dimension and 1.2 mm in height. The design was exported as STL file for 3D printing. The 3D printer used was CADWorks 3D M50 with bundled software Utility version 6.3.0.t3. The resin used was master mold for PDMS resin and the settings used were Layer Thickness = 50 um, Curing time = 5 seconds, Gap Adjustment = 0.1 mm, Base layers = 1, Base Curing = 50 seconds, Buffer Layers = 8, and Power = 100%. Once the 3D printing was done, the printed object was thoroughly washed with Isopropyl alcohol (IPA) for 30 minutes twice, air-dried before UV curing for an hour and heated in an oven at 60°C for 6 hours. The material used for soft lithography was Sylgard 184 silicone elastomer kit from Dow Corning. The two parts of the kit (resin and curing agent) were mixed at 10:1 ratio by weight, mixed thoroughly and placed in a desiccator under vacuum to remove excess air bubbles. The prepared mixture was then poured into the 3D-printed mold, taking care not to cover the four pillars, and cured in an oven at 80°C for an hour until completely cured. Then the PDMS layer was carefully removed from the mold to prevent any tearing and damage.

Thereafter, the PDMS layer and imaging chamber were placed inside an oxygen plasma cleaner (Harrick Plasma, Plasma Cleaner PDC-32G-2) with high intensity setting for a few minutes. Then immediately, they were removed from the oxygen plasma cleaner, and carefully placed the cleaned side of the PDMS layer on the center of the imaging chamber to form a tight seal.

Volumetric imaging of RPA droplets with fluorescently labeled UvsX
RPA reactions were prepared with varying concentrations of UvsX, with AZ405-labeled UvsX supplied at 2% of the final total concentration of UvsX in the reaction. One microliter samples were spotted onto an imaging chamber and placed on top of a 10x objective lens (UPLXAPO10X) of an Olympus FV3000 inverted confocal microscope equipped with FV31S-SW software. AZ405 excitation was performed

using a 405-nm laser, with fluorescence detection at 430–470 nm. Image stacks of 800 × 800 pixels (pixel size: 1.591× 1.591 μm) were acquired with a Z-interval of 7.5 μm, covering the entire depth of the imaging chamber.

The droplet segmentation workflow was developed based on PyImageJ, a Python library that enables access to ImageJ from the Python environment[5]. The workflow began by stacking image slices into a 3D z-stack. This stack was then preprocessed using Gaussian blur stack (sigma=1) and rolling ball subtraction stack (radius=7). Next, the stack pixel was converted to 8-bit value range and thresholded using Otsu's method to create a binary mask, which was further refined through the watershed algorithm and fill any holes within them.

After segmentation, the number of droplets was determined by counting the ROI across all z-slices of the z-stack image. The putative volume of each droplets were calculated according to the minimum Feret diameter.

Volumetric imaging of RPA products in droplets with PicoGreen
The RPA reactions were stained with PicoGreen by combining 9 μL of RPA reactions with 1 μL of PicoGreen staining solution (Quant-iT PicoGreen dsDNA kit, Life Technologies) to visualize amplified DNA products. One microliter samples were spotted onto an imaging chamber and placed on top of a 10x objective lens (UPLXAPO10X). PicoGreen excitation was performed using a 488-nm laser, with fluorescence detection at 500–540 nm. Image stacks of 640 × 640 pixels (pixel size: 1.989 × 1.989 μm) were acquired with a Z-interval of 7.5 μm, covering the entire depth of the imaging chamber.

The images were reconstructed into a 3D view using Olympus FV31S-SW software. For top-view representation, the reconstructed image was cropped and zoomed to 125% to represent a 1 mm x 1 mm dimension, with small cubic grid lines (100 μm x 100 μm x 100 μm) overlaid for reference. For side-view representation, the reconstructed image was cropped and zoomed to 115% instead.

To obtain the average PicoGreen intensities of each z-slice (Fig. 2c-d), a region of interest (ROI) was manually created to encompass the droplet region located within the 1 mm x 1 mm well. The Graph Analysis menu in Olympus FV31S-SW software was used to perform a series analysis, and the average PicoGreen intensities for each z-slice were exported for further quantitative analysis.

To obtain droplet-level PicoGreen intensities, the same droplet segmentation workflow as fluorescently labeled UvsX was used with minor modifications of Gaussian blur stack (sigma=0.75). After segmentation, the number of droplets was determined by counting the ROI across all z-slices of the z-stack image. The mean intensity of PicoGreen inside the droplet and the minimum Feret diameter were also measured across all z-slices of the z-stack image for each UvsX concentration. The putative volume of each droplets were calculated according to the minimum Feret diameter.

Dual-color volumetric imaging of AZ405-labeled UvsX and PicoGreen in RPA droplets

The RPA reactions with AZ405-labeled UvsX were prepared and stained with PicoGreen to visualize amplified DNA products. Imaging was performed on the FV3000 confocal microscope equipped with dual excitation lasers at 405 nm (for UvsX) and 488 nm (for PicoGreen). Z-stack imaging was conducted by capturing optical slices from the bottom of the well to a height of 180 µm. A total of 25 slices were acquired at 7.5 µm intervals, with each slice captured separately for the 405 nm and 488 nm excitation channels to ensure distinct and accurate fluorescent signal acquisition. The resolution for each slice was set at 640x640 pixels. The Z-stack images were processed using Olympus FV31S-SW software to reconstruct a 3D view. For top-view representation, the reconstructed image was cropped and zoomed to 125% to represent a 1 mm x 1 mm dimension, similar to the settings used for PicoGreen imaging.

Fluorescence microscopy of RPA with high-magnification objective lens

Imaging of 15-µL RPA reactions was carried out using a 35 mm Glass base dish (IWAKI) in a buffer solution consisting of 50 mM Tris-HCl pH 7.5, 100 mM potassium acetate, 5% (w/v) PEG20000, 2 mM DTT, and 14 mM magnesium acetate. RPA components were freshly mixed before imaging, which happened within 2 minutes after mixing. The experiments were conducted at either room temperature or a specified temperature using an inverted Olympus Fluoview FV3000 confocal microscope and either a 60× or 100× oil immersion objective. Fluorescence quantification of resulting images was performed using ImageJ software[6].

Fluorescence Recovery After Photobleaching (FRAP)

For *in vitro* FRAP, 26 µM Gp32 and 6 µM UvsX was allowed to phase separate in the presence of RPA buffer (50 mM Tris-HCl pH 7.5, 100 mM potassium acetate, 5% (w/v) PEG20000, 2 mM DTT, 14 mM magnesium acetate). All the reaction mixtures contained 2% and 6% labeled protein/labeled protein for UvsX labeled with AZDye™ 405 and for Gp32 labeled with AZDye™ 568, respectively, to minimize the effect of protein labeling on LLPS. Imaging of 15-µL samples was carried out using a 35 mm Glass base dish (IWAKI). After droplet formation was confirmed by fluorescence microscopy, the dishes containing the droplets were subjected to FRAP experiments.

FRAP studies were performed using a laser scanning confocal microscope (Olympus Fluoview FV3000) with a 100X oil immersion objective. The selected region of interest (ROI-1, ~5 µm) was bleached with 90 % laser power. All the measurements were performed at room temperature and at triplicates. After acquiring an image before bleaching, the fluorescence recovery was monitored. The images were corrected for laser bleaching by selecting a fluorescent region outside the ROI (reference region, ROI-2). The images were obtained with a frame size of 512 pixels x 512 pixels with 16-bit-dept, and later analyzed with ImageJ.

FRAP data analysis

Fluorescence intensities were measured from three distinct regions of interest (ROIs) with constant radii. These regions include the actual bleached region (ROI-1), a reference region on the same droplet (ROI-2) but at a different location to account for passive bleaching caused by laser exposure, and a region outside the droplet to correct for background fluorescence intensity (ROI-3).

Fluorescence recovery curves were generated by analyzing total intensity fluorescence values within the ROI for each frame, with corrections applied for background and laser scanning bleaching. Full-scale normalization was employed, additionally correcting for differences in bleaching depth by subtracting the intensity of the first post-bleach image in ROI-1. The formula defining full-scale normalization is:

$$I\ (t)_{double\ norm} = \left(\frac{I(t)_{ROI-1}}{I(t)_{pre-bleach\ ROI-1}}\right)\left(\frac{I(t)_{pre-bleach\ ROI-2}}{I(t)_{ROI-2}}\right)$$

$$I(t)_{norm}^{full\ scale} = \frac{I(t)_{double\ norm} - I(t_{post-bleach})_{double\ norm}}{1 - I(t_{post-bleach})_{double\ norm}}$$

To achieve fully normalized measurements of fluorescence intensity, we performed the following steps: firstly, subtracting the fluorescence intensity of the background (ROI-3) from both the bleached droplet (ROI-1) and the reference droplet (ROI-2). Further normalization was carried out by dividing the fluorescence intensities of the bleached droplet and the reference droplet by their respective pre-bleach fluorescence intensities, setting the pre-bleach time point to 1 (double normalization[7]). To correct for photobleaching resulting from prolonged exposure during time-coursed measurements, fluorescence intensities of the bleached droplet were divided by the fluorescence intensities of a reference droplet. This normalization step aimed to account for variations in bleaching depth. The fluorescence intensities, following these normalization procedures, were plotted against time[7, 8].

Line profile analysis of *Bsu* Pol fluorescence intensity in droplets

Confocal images of droplets were analyzed using ImageJ. The images were first converted to 8-bit grayscale format (*Image → Type → 8-bit*). A straight line measuring 16 µm was manually drawn across each droplet using the Line Tool, ensuring that the line was centered on the droplet. The line intensity profile for each droplet was extracted using the *Analyze → Plot Profile* function, and the resulting data were exported as CSV files for further analysis.

To standardize the line intensity profiles, the position values were normalized to a scale of [-1, 1]. The normalization range was defined as the region between the first and last positions where the intensity exceeded a threshold of 35. Within this range, intensity profiles from all droplets were averaged to generate a representative curve. The averaged curve was smoothed using Locally Weighted Scatterplot Smoothing (LOWESS), which considered 15% of the data points for each estimation.

Cultured SARS-CoV-2 viral RNA extracts, serial dilutions, and RT-qPCR

SARS-CoV-2 (clinical isolate hCoV-19/Thailand/Siriraj_5/2020; GISAID accession ID: EPI_ISL_447908) was propagated in Vero E6 cells grown in MEM-E supplemented with 10% fetal bovine serum at the Department of Microbiology, Faculty of Medicine Siriraj Hospital. The culture media were collected and their RNA content extracted using a magLEAD 12gC automated extraction platform (Precision System Science, Japan) as previously described[3]. The RT-qPCR was performed using Luna Universal Probe One-Step RT-qPCR Kit (New England Biolabs) and nCoV_N1 primer-probe set (Integrated DNA Technologies) and monitored on a CFX Connect Real-Time PCR System (Bio-Rad) as previously described[3]. The research on DNA extracts from

cultured viruses was classified as non-human subject research by Human Research Protection Unit, Faculty of Medicine Siriraj Hospital, Mahidol University, and was not subjected to review by the Institutional Review Board.

References

(1) Hu, G.; Katuwawala, A.; Wang, K.; Wu, Z.; Ghadermarzi, S.; Gao, J.; Kurgan, L. flDPnn: Accurate intrinsic disorder prediction with putative propensities of disorder functions. *Nature Communications* **2021**, *12* (1), 4438. DOI: 10.1038/s41467-021-24773-7.
(2) Madeira, F.; Pearce, M.; Tivey, A. R. N.; Basutkar, P.; Lee, J.; Edbali, O.; Madhusoodanan, N.; Kolesnikov, A.; Lopez, R. Search and sequence analysis tools services from EMBL-EBI in 2022. *Nucleic Acids Res.* **2022**, *50* (W1), W276-W279. DOI: 10.1093/nar/gkac240 PubMed.
(3) Patchsung, M.; Homchan, A.; Aphicho, K.; Suraritdechachai, S.; Wanitchanon, T.; Pattama, A.; Sappakhaw, K.; Meesawat, P.; Wongsatit, T.; Athipanyasilp, A.; et al. A Multiplexed Cas13-Based Assay with Point-of-Care Attributes for Simultaneous COVID-19 Diagnosis and Variant Surveillance. *Crispr j* **2023**, *6* (2), 99-115. DOI: 10.1089/crispr.2022.0048 From NLM.
(4) Patchsung, M.; Jantarug, K.; Pattama, A.; Aphicho, K.; Suraritdechachai, S.; Meesawat, P.; Sappakhaw, K.; Leelahakorn, N.; Ruenkam, T.; Wongsatit, T.; et al. Clinical validation of a Cas13-based assay for the detection of SARS-CoV-2 RNA. *Nature Biomedical Engineering* **2020**, *4* (12), 1140-1149. DOI: 10.1038/s41551-020-00603-x.
(5) Rueden, C. T.; Hiner, M. C.; Evans, E. L.; Pinkert, M. A.; Lucas, A. M.; Carpenter, A. E.; Cimini, B. A.; Eliceiri, K. W. PyImageJ: A library for integrating ImageJ and Python. *Nature Methods* **2022**, *19* (11), 1326-1327. DOI: 10.1038/s41592-022-01655-4.
(6) Schneider, C. A.; Rasband, W. S.; Eliceiri, K. W. NIH Image to ImageJ: 25 years of image analysis. *Nat. Methods* **2012**, *9* (7), 671-675. DOI: 10.1038/nmeth.2089.
(7) Phair, R. D.; Gorski, S. A.; Misteli, T. Measurement of dynamic protein binding to chromatin in vivo, using photobleaching microscopy. *Methods Enzymol.* **2004**, *375*, 393-414. DOI: 10.1016/s0076-6879(03)75025-3 From NLM.
(8) Ellenberg, J.; Siggia, E. D.; Moreira, J. E.; Smith, C. L.; Presley, J. F.; Worman, H. J.; Lippincott-Schwartz, J. Nuclear Membrane Dynamics and Reassembly in Living Cells: Targeting of an Inner Nuclear Membrane Protein in Interphase and Mitosis. *J. Cell Biol.* **1997**, *138* (6), 1193-1206. DOI: 10.1083/jcb.138.6.1193 (acccessed 12/2/2023). Sprunger, M. L.; Jackrel, M. E. Monitoring condensate dynamics in S. cerevisiae using fluorescence recovery after photobleaching. *STAR Protocols* **2022**, *3* (3), 101592. DOI: https://doi.org/10.1016/j.xpro.2022.101592.
